# Surface Engineering Enabled Capacitive Gas‐Phase Water Molecule Sensors in Carbon Nanodots

**DOI:** 10.1002/advs.202414611

**Published:** 2025-04-26

**Authors:** Jin‐Xu Qin, Cheng‐Long Shen, Wu‐You Zhang, Yuan Deng, Shou‐Long Lai, Chao‐Fan Lv, Hang Liu, Ying‐Jie Zhang, Lan Liu, Lei Li, Xi‐Gui Yang, Chong‐Xin Shan

**Affiliations:** ^1^ Henan Key Laboratory of Diamond Optoelectronic Material and Devices Key Laboratory of Integrated Circuit Ministry of Education School of Physics Zhengzhou University Zhengzhou 450052 China; ^2^ School of Computational Science and Electronics Hunan Institute of Engineering Xiangtan 411104 China; ^3^ Institute of Quantum Materials and Physics Henan Academy of Sciences Zhengzhou 450046 China

**Keywords:** capacitive sensor, carbon nanodots, molecular affinity, surface engineering

## Abstract

Gas‐phase water molecule sensors are essential in scientific, industrial, and environmental applications, playing a crucial role in ensuring human safety, monitoring pollution, and optimizing processes. However, developing gas‐phase water sensors with high sensitivity remains a significant challenge. Herein, the effect of molecular adsorption on capacitive response is explored, and a facile surface engineering strategy to achieve sensitive carbon nanodots (CDs)‐based sensors for H_2_O is demonstrated.hydrophilic raw precursor is utilized to prepare the hydrophilic CDs and further employ these CDs as active media in the capacitive sensors, demonstrating how surface adsorption influences the capacitive response to H_2_O molecules. By applying surface engineering, the molecular affinity potential of CDs is regulated, resulting in sensors that exhibit a broad detection range from 11% to 98% relative humidity (RH), with a remarkable sensitivity of 3.3 × 10^5^ pF/RH and an impressive response of 1.8 × 10^8^% at 98% RH. These CDs‐based sensors present great potential for applications in respiratory monitoring, information exchange, contactless recognition of finger trajectories, etc. The findings unveil the unique influence of molecular affinity on capacitive response, opening new avenues for the design and applications of highly sensitive molecular sensors.

## Graphical abstract

A surface engineering strategy is developed to tune the molecular affinity of carbon nanodots (CDs), leading to capacitive sensors with an impressive detection range from 11% to 98% relative humidity (RH). These sensors demonstrate high sensitivity, with 3.3 × 10^5^ pF/RH, and an exceptional response of 1.8 × 10^8^ % at 98% RH. This approach unlocks new possibilities for CDs in practical applications, such as respiratory monitoring, contactless recognition of finger trajectories, and environmental sensing. The findings offer significant potential for future molecular sensor designs and wide‐ranging industrial applications.

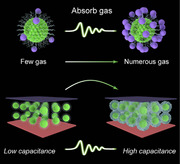

## 1 Introduction

The highly sensitive and reliable humidity monitoring is critical in the fields of environmental monitoring, healthcare applications, industrial processes, preservation of artifacts and electronics, etc.^[^ ^1^, ^2^ ^]^ To date, the conventional humidity sensors are established with optical, thermal, mass, and resistance responses under different relative humidity, and thus there are still various shortcomings in different applications.^[^ ^3^, ^4^, ^5^, ^6^, ^7^, ^8^, ^9^ ^]^ Generally, the optical humidity sensors are always bulky and expensive, the thermal humidity sensors often have a narrow measurement range, the mass‐based humidity sensors generally exhibit low sensitivity and require complex operations, the resistive humidity sensors always suffer from the low stability and reliability from Joule heating. In contrast, capacitive sensors, with their facile structure, high sensitivity, and excellent dynamic response, offer the essential capabilities to achieve precise detection and quantification of force, heat, light, electric, and magnetic field, endowing the applications of environmental safety, air quality monitoring, industrial process control, and healthcare diagnostics.^[^ ^10^, ^11^, ^12^, ^13^, ^14^ ^]^ Typically, capacitive sensors are established with two separate electrodes and responsive media, whose dielectric constant can vary when the environment condition changes.^[^ ^10^, ^13^ ^]^ With the inherent advantages of excellent environment tolerance, high sensitivity, low‐cost, easy preparation, the capacitive sensors have drawn extensive attention in molecular identification and detection. In recent years, capacitive water molecular sensors, especially humidity sensors, play a crucial role in human safety, monitoring pollution, and optimizing processes, ensuring the essential scientific, industrial, and environmental requirements.^[^ ^12^, ^15^ ^]^ Nevertheless, due to the limitations of responsive media, the development of capacitive water sensors with wide‐range and sensitive responses still remains a considerable challenge. Therefore, developing capacitive media with improved sensitivity, selectivity, and stability of water active response is still crucial.

Nanomaterials, featured by their small size, large specific surface, and abundant functional groups, have shown great promise as responsive materials in various sensors, enabling the determination analysis of gas molecules in diverse environments.^[^ ^16^, ^17^, ^18^ ^]^ To date, nanomaterials, such as carbon nanomaterials, ZnO, h‐BN, and metal–organic frameworks, have been extensively utilized as responsive media in capacitive or resistance sensors for detecting gases like NO_2_, H_2_O, CO_2,_ and NH_3_.^[^ ^15^, ^18^, ^19^, ^20^ ^]^ Among these materials, carbon nanodots (CDs),^[^ ^21^, ^22^, ^23^, ^24^, ^25^, ^26^ ^]^ a novel type of 0D carbon materials, have presented promising potential in gas sensors.^[^ ^27^ ^]^ Their large specific surface area and tunable surface functional groups make them ideal for achieving novel optical or electrical responses through controlled interactions with gas molecules. This versatility allows for the customization of sensing characteristics, enabling high selectivity and sensitivity across a wide range of gas species. However, research on CDs in gas sensing remains limited, underscoring the need for the development of high‐sensitivity CD‐based gas molecular sensors.

In this work, we investigate the effect of molecular adsorption on capacitive response and propose a facile surface engineering strategy to achieve wide‐range and sensitive CD‐based sensors for H_2_O detection. We utilize hydrophilic raw precursor to prepare the hydrophilic CDs and further employ these CDs as active media in capacitive sensors, demonstrating that the surface adsorption from nano‐scaling can improve the capacitive response to H_2_O molecules. With the surface engineering to regulate the molecules affinity potential of CDs, a broad and sensitive capacitive response for H_2_O can be realized in the CDs‐based capacitive sensor, resulting in sensors that exhibit a broad detection range from 11% to 98% relative humidity (RH), with a remarkable sensitivity of 3.3 × 10^5^ pF/RH and an impressive response of 1.8 × 10^8^% at 98% RH. With these sensitive capacitive sensors, we differentiate between nasal and oral breathing modes for respiratory monitoring, establish a signal transmitter for information exchange, and develop a large‐scale sensor array for contactless recognition of finger trajectories. These findings reveal the unique molecular affinity effect on capacitive response and pave the way for innovative designs and advanced applications of highly sensitive molecular sensors.

## 2 Results and Discussion

**Figure 1:**
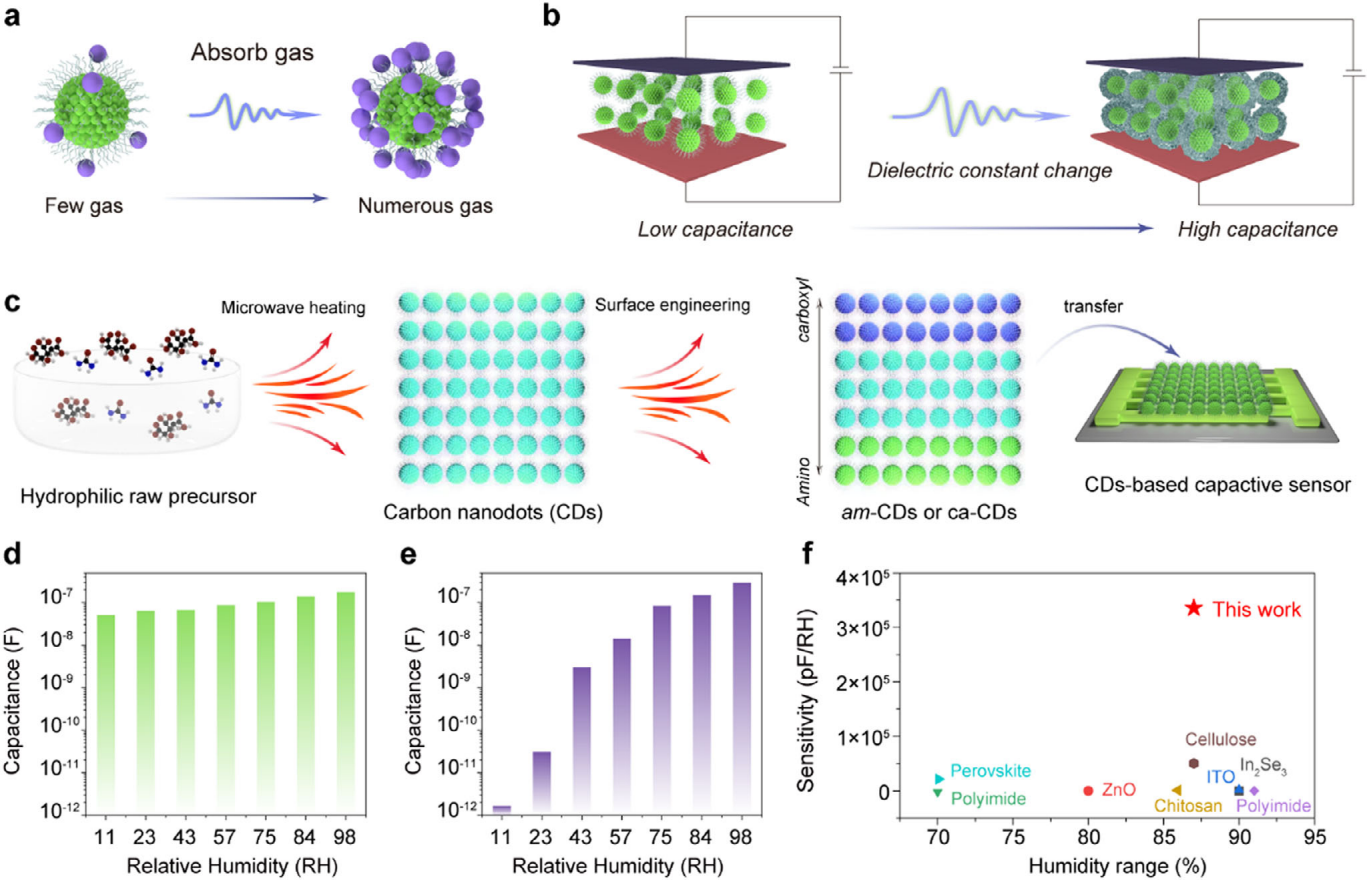
a) Schematic illustration of the molecular adsorption effect of nanoparticles. b) Schematic illustration of the relationship between capacitance and molecular adsorption effect. c) Schematic illustration of the preparation and surface engineering of hydrophilic CDs for capacitive sensor. d) Capacitance versus RHs of the precursor‐based sensor. e) Capacitance versus RHs of the surface‐engineered CD‐based sensor. f) The sensitivity of the surface‐engineered CD‐based humidity sensor in comparison with other humidity‐sensitive materials.

Nanoparticles, due to their small size, large specific surface, and abundant functional groups, can effectively adsorb various gas molecules through physical or chemical interactions (**Figure** **1**a).^[^ ^16^, ^18^ ^]^ When gas molecules are adsorbed on the nanoparticles, the dielectric constant of the nanoparticle changes, leading to a detectable variation in capacitance (Figure 1b), endowing a dynamical capacitive response to gas types and concentrations. As a proof, we employed the hydrophilic CDs as an active medium to selectively adsorb H_2_O molecules, thereby creating a high‐performance humidity sensor. As shown in Figure 1c, the CDs are prepared with the hydrophilic raw precursors of citric acid and urea via facile microwave heating and further transferred to the capacitive sensor as responsive media.^[^ ^28^, ^29^ ^]^ With the surface engineering to regulate H_2_O molecular adsorption capability, the as‐prepared capacitive sensor presents a tunable capacitive response. In comparison with the initial raw precursors (Figure S1, Supporting Information), the obtained CDs‐based capacitive sensor presents a variation of nearly five orders of magnitude in capacitance when the RH alters from 11% to 98% (Figure 1d,e), demonstrating that the surface adsorption from nano‐scaling can improve the capacitive response to H_2_O molecules. As a result, the as‐prepared CDs‐based capacitive sensor possesses a broad detection range from 11% to 98% relative humidity (RH), with a remarkable sensitivity of 3.3 × 10^5^ pF/RH at 98% RH, which is the best performances among capacitive humidity sensors (Figure 1f).^[^ ^30^, ^31^, ^32^, ^33^, ^34^, ^35^, ^36^, ^37^ ^]^ The performances of these CDs‐based sensors significantly support the hypothesis of the molecular affinity effect toward capacitive response and highlight the unique structure of CDs for the gas sensors.

**Figure 2:**
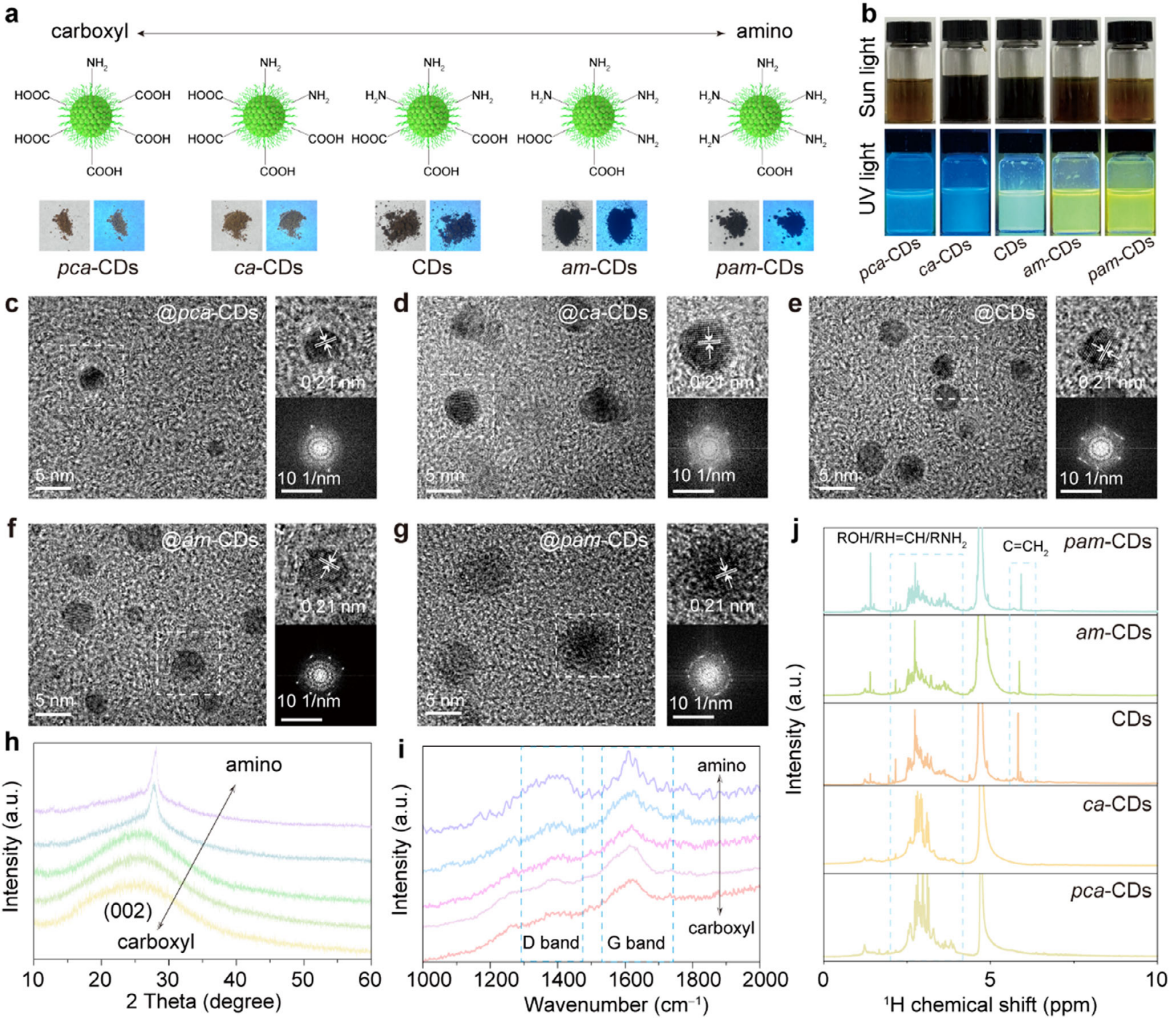
a) Schematic illustration of the surface engineering and the as‐prepared *polycarboxyl*‐CDs (*pca*‐CDs), *carboxyl*‐CDs (*ca*‐CDs), CDs, *amino*‐CDs (*am*‐CDs), *polyamino*‐CDs (*pam*‐CDs) powder under sunlight and 365 nm UV lamp. b) Optical photographs of the corresponding CDs aqueous solution under sunlight and 365 nm UV lamp. c–g) TEM, high‐resolution TEM images, and selected area electron diffraction (SAED) pattern of the corresponding CDs. h) XRD patterns of the corresponding CDs. i) Raman spectra of the corresponding CDs. j) ^1^H NMR spectra of the corresponding CDs.

As illustrated in **Figure** **2**a, we employed a surface engineering strategy to regulate the skeleton structure and surface functional groups of the hydrophilic CDs. The as‐prepared *polycarboxyl*‐CDs (*pca*‐CDs), *carboxyl*‐CDs (*ca*‐CDs), CDs, *amino*‐CDs (*am*‐CDs), *polyamino* CDs (*pam*‐CDs) can be prepared by tuning the mass ratio of citric acid and urea via the microwave‐assisted heating method (Figure S2, Supporting Information).^[^ ^28^, ^29^ ^]^ The obtained CDs powder presents a black color under sunlight and 365 nm UV light and can be easily dispersed in aqueous solutions with bright fluorescence under a 365 nm UV lamp (Figure 2b), demonstrating the well hydrophilicity of the CDs. Figure S3 (Supporting Information) shows the UV–vis absorption spectra for these CDs, and the absorption bands ≈330–340 nm are attributed to the π → π* transition of the core, while bands ≈400–408 nm are assigned to the n → π* transition of the surface state.^[^ ^38^, ^39^ ^]^ Transmission electron microscopy (TEM) images display similar particle size across all the CD types, and high‐resolution TEM (HRTEM) images reveal a lattice spacing of 0.21 nm, corresponding to the (100) plane of graphitic carbon (Figure 2c–g), as confirmed by the selected area electron diffraction (SAED) patterns shown in the insets of Figure 2c–g.^[^ ^40^, ^41^ ^]^ The X‐ray diffraction (XRD) patterns of the *pca*‐CDs, *ca*‐CDs, and CDs exhibit a broad peak at 26°, which can be attributed to the (002) plane of graphitic structures.^[^ ^41^, ^42^ ^]^ In contrast, the *am*‐CDs and *pam*‐CDs display a prominent peak at 27.6°, originating from the (110) plane of *β*‐C_3_N_4_ (Figure 2h), likely due to the phase‐changes from increased urea contents in the precursors.^[^ ^27^, ^39^ ^]^ As the urea content in the precursor increases, the core structure of the synthesized CDs transitions from graphite to sp^3^‐hybridized carbon nitride, resulting in the appearance of *β*‐C_3_N_4_ peaks.^[^ ^43^, ^44^, ^45^, ^46^ ^]^ Meanwhile, the Raman spectra of these CDs (Figure 2i) confirm their similar crystalline, with characteristic G and D bands at 1580 and 1350 cm^−1^, respectively.^[^ ^23^, ^29^ ^]^ In the ^1^H nuclear magnetic resonance (NMR) spectra (Figure 2j), the signals of ROH/RH = CH/RNH_2_ and C = CH_2_ can be observed in these CDs, verifying their similar sp^2^/sp^3^ carbon skeleton structures and surface groups.^[^ ^47^, ^48^ ^]^

**Figure 3:**
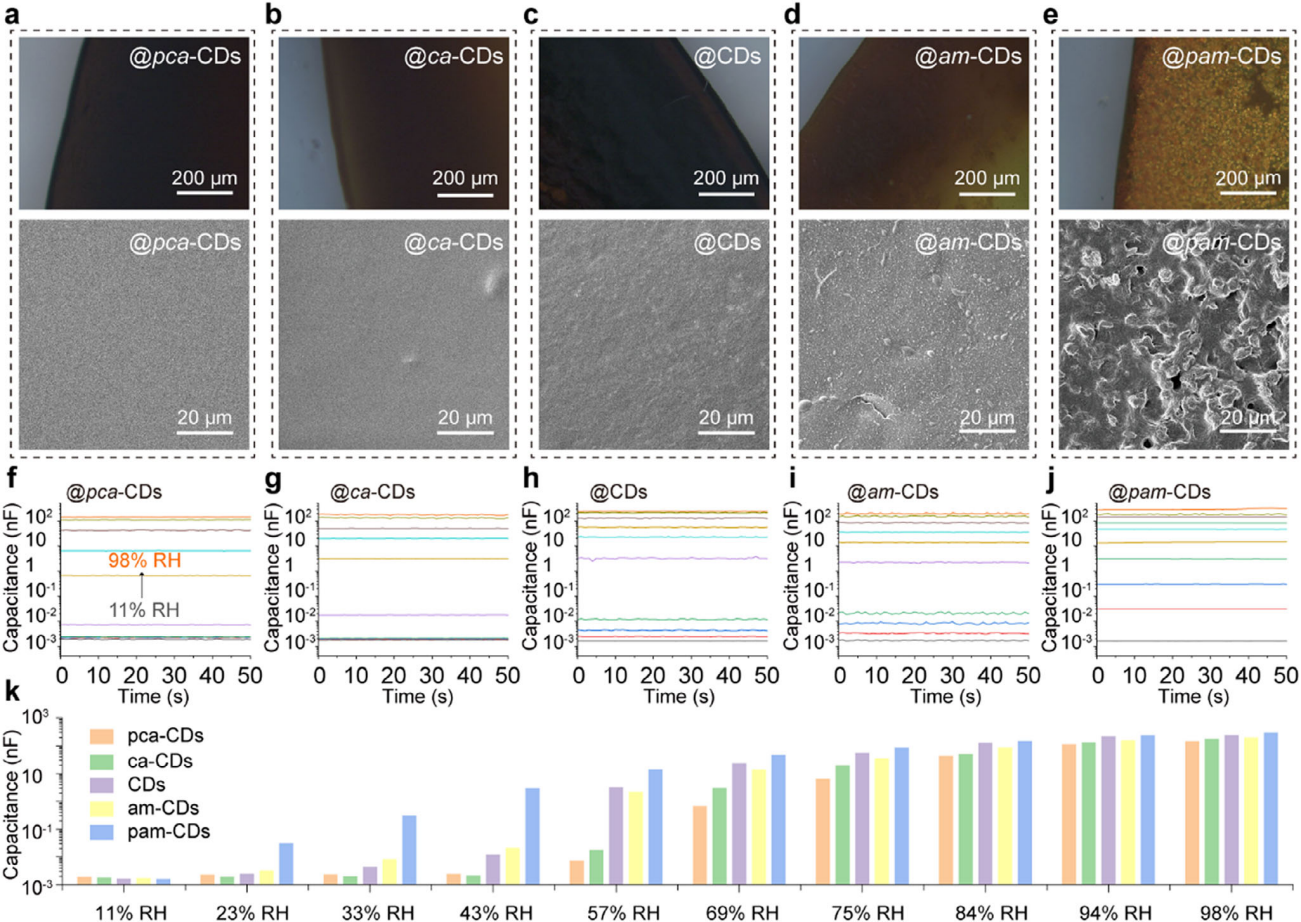
Microscopic image and SEM images of the a) *pca*‐CDs, b) *ca*‐CDs, c) CDs, d) *am*‐CDs and e) *pam*‐CDs. *C‐t* curves of the capacitive sensors prepared with f) *pca*‐CDs, g) *ca*‐CDs, h) CDs, () *am*‐CDs and j) *pam*‐CDs under different RHs. k) Capacitance response of the capacitive sensors prepared with these five CDs under different RH.

Given their similar size, morphology, and skeleton structure, we investigated the capacitive response of these CDs in capacitive sensors. As illustrated in **Figure** **3**a–e, the films of different CDs display varied stacking patterns, as shown in the photomicrographs and scanning electron microscopy (SEM) images. Notably, the *pca*‐CDs, *ca*‐CDs, CDs, and *am*‐CDs films exhibit smooth surfaces, while the *pam*‐CDs exhibit rough surface. This variation in stacking likely influences gas adsorption, leading to different capacitive responses. These CDs were employed to fabricate the sensor (Figure S4, Supporting Information), and their response to humidity change was tested. As a result, the capacitance‐time (*C‐t*) curves of these CDs‐based sensors illustrate marked differences in capacitive response to the H_2_O molecules (Figure 3f,j), with devices showing significant capacitance increases as the relative humidity rises from 11% to 98%. Remarkably, capacitance change by nearly five orders of magnitude was observed, highlighting the substantial potential for humidity sensing. While capacitance changes are minimal for *pca*‐CDs, *ca*‐CDs, CDs, and *am*‐CDs at lower humidity levels (below ≈50% RH), the *pam*‐CDs sensor exhibits a consistent increase in capacitance across the entire humidity range (Figure 3k), implying its superior sensing capabilities. The variation in the adsorption and desorption equilibrium of H_2_O molecules on these CDs in a free environment can lead to different capacitive responses. At low humidity, the varying surface affinities and desorption behaviors determine different adsorption–desorption equilibria, leading to the distinct capacitive responses from these CDs. At high humidity, the similar size of CDs can contribute to the similar surface energy and saturated H_2_O adsorption, resulting in a similar capacitive response (Figure S5, Supporting Information).

**Figure 4:**
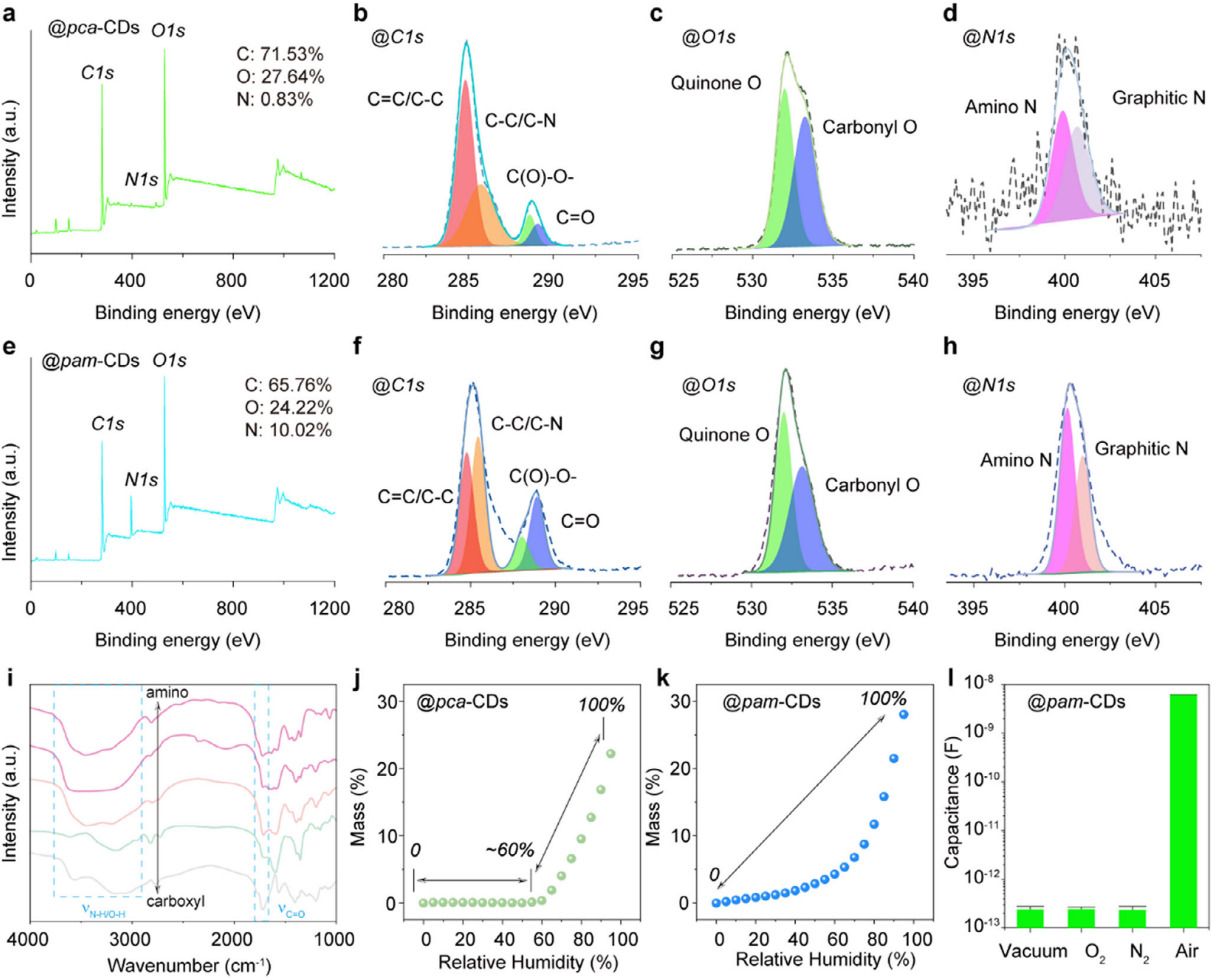
a) Full XPS survey of the *pca*‐CDs. High‐resolution b) C1s spectra, c) O1s spectra d) N1s spectra of the *pca*‐CDs. e) Full XPS survey of the *pam*‐CDs. High‐resolution f) C1s spectra, g) O1s spectra, and h) N1s spectra of the *pam*‐CDs. i) FT‐IR spectra of the *pca*‐CDs, *ca*‐CDs, CDs, *am*‐CDs, and *pam*‐CDs. j) DVS curves of the *pca*‐CDs. k) DVS curves of the *pam*‐CDs. l) Capacitive response of the *pam*‐CDs‐based sensor in vacuum, oxygen (O_2_), nitrogen (N_2_), and air.

To elucidate the discrepant capacitive response further, we conducted additional tests on these CDs. X‐ray photoelectron spectroscopy (XPS) analysis reveals that all CDs contain similar elements: C (284.8 eV), O (527.7 eV), and N (395.8 eV) (**Figures** **4**a,e; Figure S6, Supporting Information). High‐resolution C1s spectra can be deconvoluted into C═C/C─C, C─C/C─N, C(O)‐O─ and C═O (Figure 4b,f).^[^ ^49^, ^50^ ^]^ High‐resolution O1s spectrum reveals the presence of C─O and O═C─O groups (Figure 4c,g),^[^ ^21^ ^]^ while the high‐resolution N1s spectrum shows signals for amino N and graphitic N (Figure 4d,h),^[^ ^51^, ^52^, ^53^ confirming a similar sp^2^‐hybrid carbon skeleton with carbonyl and amino groups. Compared to the *pca*‐CDs, the *pam*‐CDs present similar O content but lower carbonyl O and higher N content, suggesting that the amino group enhances H_2_O adsorption. Fourier‐transform infrared (FT‐IR) spectra of these CDs exhibit characteristic peaks at 1639, 1400, 1048, and 3442 cm^−1^, associated with C═O, C─N, C─O, and particularly hydrophilic N─H/O─H bonds (Figure 4i), further supporting their similar functional groups.^[^ ^54^, ^55^, ^56^ ^]^ Notably, the CDs synthesized with higher urea content in the precursors exhibit increased N─H/O─H peak intensities, confirmed by XPS analysis. Dynamic vapor sorption (DVS) curves reveal that both the *pca*‐CDs and *pam*‐CDs can gradually adsorb water molecules, with mass increase correlating with increasing RH (Figure 4j,k). Similar to the capacitive changes, mass increases for the *pca*‐CDs occurred only after humidity surpassed ≈60%, approving the role of molecule adsorption in the capacitive response. Moreover, the *pam*‐CDs exhibit significantly lower capacitance values in vacuum, oxygen, nitrogen, argon, carbon dioxide, hydrogen–nitrogen mixture, and hydrogen–argon mixture environments, implying that these gases do not affect the capacitive response (Figures 4l; Figure S7, Supporting Information). Therefore, it can be deduced that N‐related surface groups on the CDs play a crucial role in water molecule adsorption, resulting in the observed differences in capacitive response.

**Figure 5:**
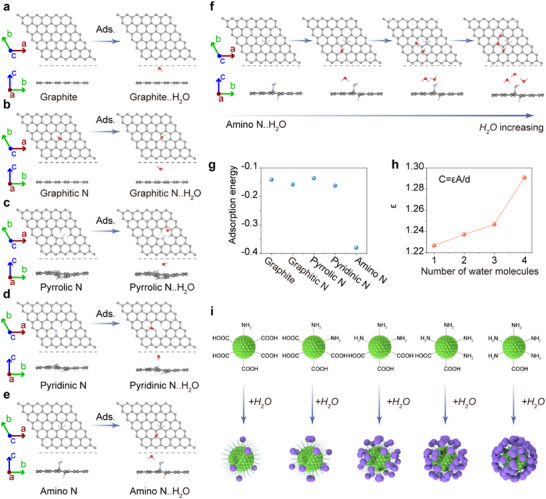
Schematic illustration of H_2_O molecule adsorption on the surface of a) graphite, b) graphitic N, c) pyrrolic N, d) pyridinic N, and e) amino N. f) Schematic illustration of amino N‐related graphite surfaces with 0, 1, 2, and 3 H_2_O molecules. (g) Calculated adsorption energy for graphite, graphitic N, pyrrolic N, pyridinic N, and amino N. h) Dielectric constant versus the number of H_2_O molecules. i) Schematic illustration of the capacitive response mechanism in the CD‐based sensors.

To understand how the surface group influences the capacitive response further, we employed density functional theory (DFT) to analyze the relationship between the surface groups and capacitance. As depicted in **Figures** **5**a–e and Figure S8 (Supporting Information), we established the N‐related structures such as graphite, graphitic N, pyrrolic N, pyridinic N, and amino N with one adsorbed H_2_O molecule. The corresponding adsorption energies for these surfaces are measured as follows: −0.141 eV for graphitic N, −0.158 eV for graphitic N, −0.136 eV for pyrrolic N, −0.162 eV for pyridinic N, and −0.38 eV for amino N (Figure 5g). Among these surfaces, amino N exhibits the lowest adsorption energy, indicating the highest affinity potential for H_2_O molecules (Figure 5g).^[^ ^57^ ^]^ We then compared the permittivity (*ε*) of amino N‐related graphite surfaces with varying H_2_O molecules adsorption (0 to 3 molecules) (Figures 5f; Figure S9, Supporting Information). Generally, the relationship between permittivity and capacitance in a capacitor can be defined by the following equation^[^ ^31^, ^32^ ^]^:

(1) $$C=\frac{\varepsilon A}{d}\;$$

where *C* is capacitance, *ε* is permittivity, *A* is plate area, and *d* is plate distance. The calculations reveal that *ε* increases as the number of adsorbed H_2_O molecules increases, suggesting that amino N significantly contributes to H_2_O adsorption, which in turn alters capacitance. This results in the wide‐ranging and sensitive capacitive response observed in the *pam*‐CDs (Figure 5g,h). Summarizing these results, it can be concluded that the varying surface groups of CDs affect their affinity for H_2_O, leading to distinguishable permittivity under the same RH (Figure 5i) and consequently diverse capacitive responses. To further investigate the sensing mechanism of the pam‐CDs‐based capacitive humidity sensor, we measured their electrical conductivity under different RHs. The surveys reveal that, the conductivity of the pam‐CDs sensor gradually increases as humidity increases, indicating the increased number of surface ions after rising RH (Figure S10, Supporting Information). These results imply that the ionic capacitance effect (electrical double layer, EDL effect) may contribute to the several orders of magnitude change in the pam‐CDs‐based capacitive sensor. To further verify this hypothesis, we have introduced different salts into the pam‐CDs to increase the number of ions. The capacitance measurements revealed that the capacitance of the salt‐doped sensor was higher than that of the original pam‐CDs‐based sensor at both low humidity (11% RH) and high humidity (98% RH) (Figure S11, Supporting Information). It can be easily deduced that the anions can migrate toward the positively charged electrode and the cations can move toward the negatively charged electrode under an applied voltage. Thus, as the number of mobile ions increases with humidity, the capacitance of the sensor significantly increases, leading to its superior humidity‐sensing performance (Figure S12, Supporting Information). These result strongly supports the crucial role of ion concentration in determining the sensor's output capacitance, further confirming that the several orders of magnitude change in capacitance with humidity originate from the ionic capacitance (electrical double layer, EDL) effect.

**Figure 6:**
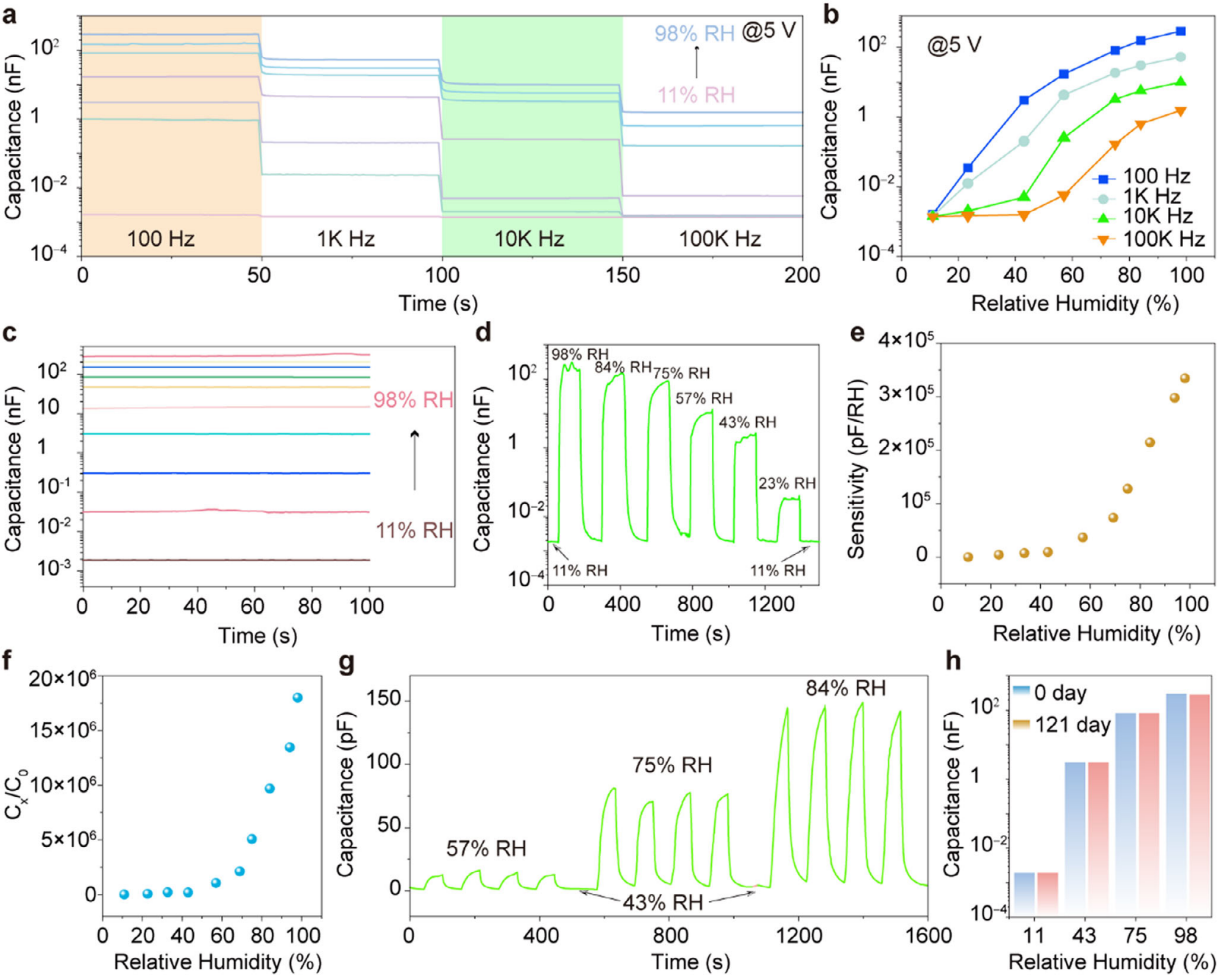
a) *C‐t* curves of the *pam*‐CD‐based sensor under different frequency and RH. b) The corresponding capacitance of the *pam*‐CD‐based sensor under different frequency and RHs. c) *C‐t* curves of the *pam*‐CD‐based sensor @100 Hz/5V under different RHs. d) Response and recovery curves of the *pam*‐CD‐based sensor under different RHs @100 Hz/5V. e) Sensitivity versus RH of the *pam*‐CD‐based sensor. f) Response versus RH of the *pam*‐CD‐based sensor. g) Repeatability of the *pam*‐CD‐based sensor from 43% RH to 57%, 75% and 84% RH. h) Stability of the *pam*‐CD‐based sensors under different RHs for 30 days.

With this sensitivity to humidity, we performed a through‐going evaluation of the *pam*‐CDs in H_2_O sensing. As illustrated in **Figure** **6**a,b, the *C‐t* curves and capacitance values of the *pam*‐CDs sensor can work at different frequencies and RHs. As frequency increases, the capacitive response gradually diminishes, likely due to the slower polarization speed of H_2_O molecules, which limits their contribution to capacitance in high‐frequency electric fields. In addition, the CDs‐based sensor presents nearly the same performance after humidity stimulation, implying the little influence of frequency and humidity on the sensors' response and recovery time (Figure S13, Supporting Information). Figure 6c shows the *C‐t* curves of the *pam*‐CDs sensor at 100 Hz under various RHs. As humidity increases from 11% RH to 98% RH, the capacitance rises dramatically from 1.6 pF to 291 nF, demonstrating a remarkable five orders of magnitude change. The sensor also presents exceptional response and recovery characteristics across a broad humidity range (Figure 6d). Sensitivity (S) and responsivity (R) are pivotal metrics to evaluate the sensor performance. The sensitivity can be defined as follows^[^ ^34^, ^36^ ^]^:

(2) $$S=\frac{C_{x}-C_{0}}{\%RH_{x}-\%RH_{0}}$$

where *C_x_* is capacitance at x% RH and *C_0_* at 11% RH. As illustrated in Figure 6e, the *pam*‐CDs sensor displays low sensitivity at low humidity, which increases significantly with increasing humidity, achieving a high sensitivity of 334560 pF/RH under 98% RH, the best performance among capacitive humidity sensors (**Table** **1**).^[^ ^30^, ^31^, ^32^, ^33^, ^34^, ^35^, ^36^, ^37^, ^58^, ^59^, ^60^, ^61^, ^62^ ^]^ The response amplitude is defined by the formula:^[^ ^36^ ^]^

(3) $$R\;\left(\%\right)=\frac{C_{x}}{C_{0}}\;*100$$

**Table 1:** The capacitive response of different nanomaterials.

Materials	S [pF/RH]	R [%]	Humidity range [RH]	Refs.
Indium Tin Oxide	7.76	N/A	5–95	30
Zirconium Phosphate	N/A	2409	10.9–91.5	58
Polyimide	22.29	N/A	20–90	31
Polyimide	82.4	N/A	6–97	32
CsPb_2_Br_5_/BaTiO_3_	21426.6	N/A	25–95	33
Chitosan‐NaCl	1017.2	∼1200	6–92	34
ZnO/Graphene Oxide	1.91	N/A	10–90	35
Cellulose nanopapers	50225	1896	7–94	36
Chitosan	N/A	10000	20–90	59
In_2_Se_3_	0.177	N/A	5–95	37
Carbon nanotube	2.22×10^6^	N/A	50–85	60
BaTiO_3_	5.75 × 10^5^	N/A	20–80	61
Phosphorus‐doped dielectric electrolyte	1.16 × 10^6^	N/A	1–95	62
*pam*‐CDs	334560	17998640	11–98	This work

As a result, the response curve follows a similar trend, reaching 17998640% under 98% RH (Figure 6f), also ranking among the best (Table 1).^[^ ^30^, ^31^, ^32^, ^33^, ^34^, ^35^, ^36^, ^37^, ^58^, ^59^, ^60^, ^61^, ^62^ ^]^ Figure 6g shows four cycles of capacitance response at various RH levels (43% to 84% RH), exhibiting consistent and reliable response with minimal fluctuations, indicating the excellent absorption and desorption behavior of *pam*‐CDs. Long‐term stability is evaluated over 121 days, with capacitance remaining nearly constant at each RH level (Figures 6h; Figure S14, S15, Supporting Information), confirming the durability and reliability of the *pam*‐CD‐based sensor. In addition, the sensor underwent nine cyclic tests, during which the capacitance was measured as the humidity increased from 11% RH to 98% RH and then decreased back to 11% RH (Figure S16, Supporting Information). The results show low hysteresis and consistent measurements across all nine tests, indicating the device's excellent stability and repeatability. In addition, the capacitance of the sensor in certain organic solvent environments is much smaller than that in a water environment (ethanol, formamide, toluene, chlorobenzene, and isopropanol), demonstrating its excellent anti‐interference ability (Figure S17, Supporting Information). These findings demonstrate the exceptional sensitivity, broad sensing range, and outstanding stability of the CD‐based humidity sensor, indicating their promising applications.

**Figure 7:**
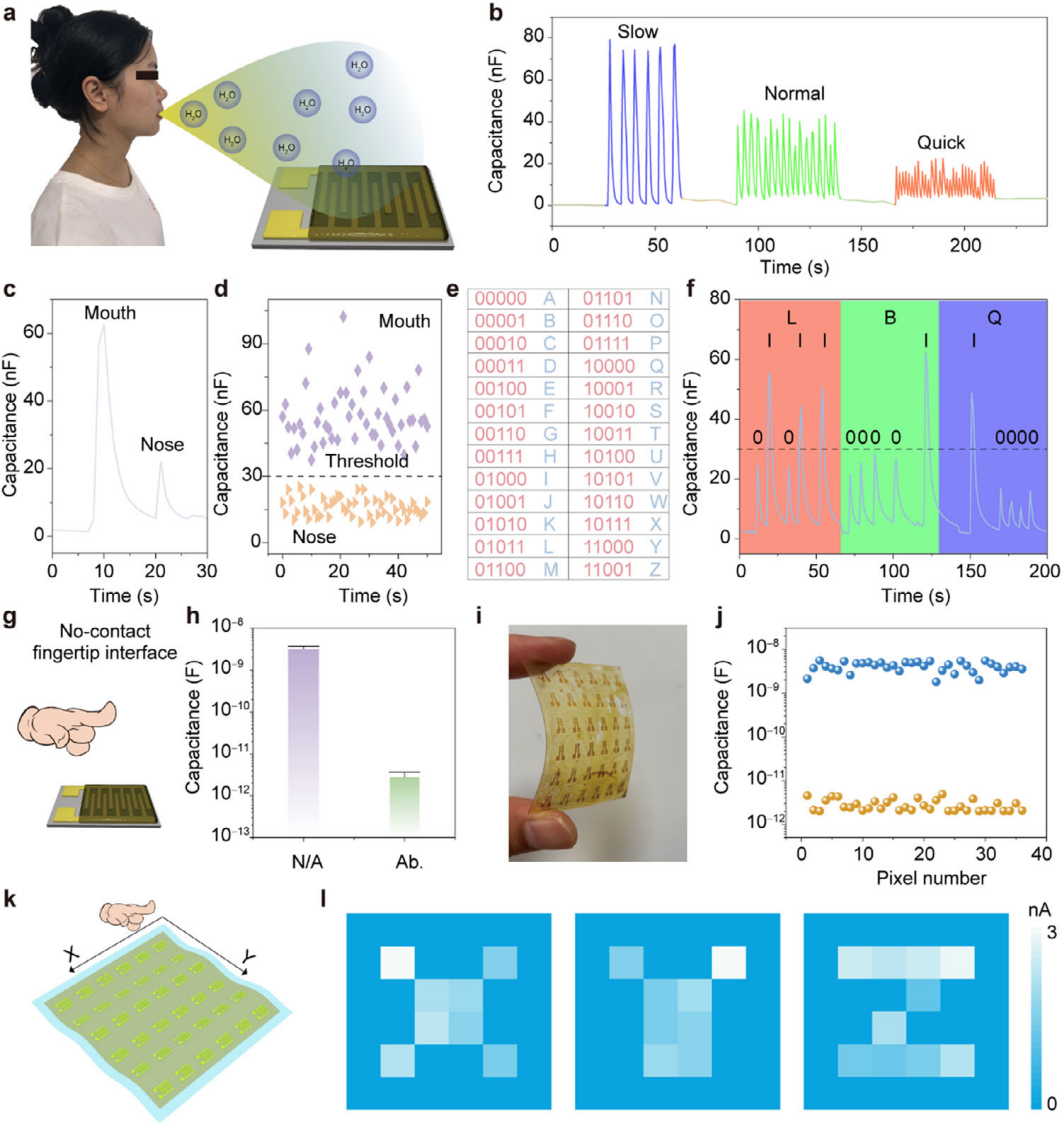
a) Schematic illustration of the human breath monitoring using the *pam*‐CD‐based sensor. b) Response curves for the sensor with quick, normal, and slow breath patterns. c) Response curve during oral and nasal exhalation. d) Statistics of peak capacitance intensity for nasal versus oral respiration, derived from 50 breaths of each type. e) A table of binary codes representing letters through combinations of 0 and 1. f) Binary encoding of the letter “L,” “B” and “Q” from breath patterns. g) Schematic of fingertip contactless application with the *pam*‐CD‐based sensor. h) Capacitance values of the sensor with and without a nearby finger. i) Photograph of the *pam*‐CD‐based sensor array. j) Capacitance values of all sensor pixels with and without a finger present. k) Schematic illustration of a finger sliding across the sensor array without contact. l) Imaging maps of shapes “X,” “Y,” and “Z” traced by a finger above the array.

Motivated by the high‐performance of the *pam*‐CD‐based sensor, we explored its application in human breath monitoring. As shown in **Figure** **7**a, during exhalation, the adsorption of water molecules on the CDs' surface increases capacitance (Figure S18, Supporting Information). Conversely, during inhalation, desorption returns capacitance to its original level (Figure S19, Supporting Information). Figure 7b illustrates reliable signals from the sensor at various breathing rates (slow, normal, and quick), with quick breathing producing faster signal changes and slow breathing resulting in longer cycles and higher intensity. These results confirm the sensor's capability for real‐time respiratory monitoring through humidity changes. Figure 7c demonstrates distinct capacitance changes during nasal versus oral exhalation, with oral breathing yielding significantly higher capacitance values due to larger air volume (Figure 7d). By setting a threshold (30 nF), we encoded breathing patterns into binary data, allowing the representation of all 26 English letters through various combinations of breathing patterns (Figure 7e). Figure 7f displays the binary data obtained from alternating nasal and oral breaths, corresponding to the letters of “L,” “B” and “Q”

Additionally, the *pam*‐CD‐based sensor can detect the humidity around a finger, typically higher than ambient levels, facilitating contactless finger tracking (Figure 7g). A significant difference in capacitance is observed with and without a nearby finger (Figure 7h), establishing a foundation for the application in contactless finger tracking. A large‐scale 6 × 6 CD‐based array is prepared (Figures 7i; Figure S20, Supporting Information), maintaining consistent capacitance levels across pixels (Figure 7j). This array enables contactless finger trajectory recognition, with varying capacitance as a finger moves across the sensors (Figures 7k; Figure S21, Supporting Information). As illustrated in Figures 7l and Figure S22–S24 (Supporting Information), the distinct trajectories (“X,” “Y” and “Z”) can be recorded by the device and the trajectory can be clearly distinguishable.

## 3 Conclusion 

In summary, surface engineering was utilized to develop sensitive capacitive humidity sensors of CDs. The CD‐based sensor exhibits exceptional performance, including a remarkable sensitivity of 3.3 × 10^5^ pF/% RH and an impressive response of 1.8 × 10^8^% at 98% RH, along with a broad detection range from 11% to 98% RH and outstanding long‐term stability. It effectively distinguishes breathing modes, serves as a signal transmitter for information exchange, and enables contactless recognition of finger trajectories through large‐scale sensor arrays. These results highlight the distinctive surface properties of CDs and pave the potential to enhance the development of gas molecular sensors.

## 4 Experimental Section

### Characterization and Measurements

Transmission electron microscopy (TEM) images were taken by a JEM2010 (JEOL, Japan) electron microscope, while scanning electron microscopy (SEM) images were characterized by a JSM‐IT200. Raman spectra were recorded using a Renishaw inVia spectrometer, and X‐ray diffraction (XRD) patterns were measured with a D/MAX‐3B diffractometer (Rigaku) with Cu K_α_ radiation. The absorption and fluorescence spectra were obtained on a Hitachi UH‐4150 UV–vis–NIR spectrophotometer and a Hitachi F‐7000spectrophotometer, respectively. Fourier‐transform infrared (FT‐IR) spectroscopy was collected using a Thermo Scientific Nicolet iZ 10 spectrometer in the range of 400–4000 cm^−1^. The chemical composition was analyzed by X‐ray photoelectron spectroscopy (XPS) using a Thermo scientific Escalab 250Xi with Al radiation. The capacitances were measured using a semiconductor parameter analyzer (Keithley 6514). Humidity environments were generated using saturated salt solution method:^[^ ^18^ ^]^ LiCl (RH 11%), C_2_H_3_KO_2_ (RH 23%), K_2_CO_3_ (RH 43%), NaBr (RH 57%), NaCl (RH 75%), KCl (RH 84%), and K_2_SO_4_ (RH 98%).

### Synthesis of the Different CDs

The CDs were synthesized using a microwave‐assisted heating method.^[^ ^28^, ^29^ ^]^ For standard CDs, 3 g of citric acid and 3 g of urea were dissolved in 20 mL of deionized water and heated in a microwave for over 10 min. After cooling to room‐temperature, a brown solid formed, which was then placed in a vacuum oven at 60 °C for 1 h to remove residual small molecules. The solid was dissolved in water and centrifuged to eliminate larger or aggregated particles. Variations for *pca*‐CDs, *ca*‐CDs, *am*‐CDs and *pam*‐CDs involved using different ratios of citric acid and urea as follows: 5 g citric acid and 1 g urea; 4 g citric acid and 2 g urea; 2 g citric acid and 4 g urea; 1 g citric acid and 4 g urea, respectively.

### Fabrication of the CDs‐Based Sensors

The sensors were fabricated by spin‐coating and natural drying the corresponding solution on a plug‐finger electrode (Figure S25, Supporting Information). Actually, a 500‐nm‐thick gold layer was first deposited onto the PET substrate via thermal evaporation, forming interdigital Au electrodes. And the finger spacing was 0.1 mm. Then, the CDs solution of different nanoparticles was dropped on the electrode and naturally dried.

### Theoretical Calculation Methods

The spin‐polarized density functional theory (DFT) calculations have been conducted on Vienna ab‐initio simulation package (VASP) to study adsorption properties of prepared materials.^[^ ^63^, ^64^ ^]^ The Projector augmented wave method with a cutoff energy of 450 eV accompanied by Perdew–Burke–Ernzerhof functional was used in the DFT calculations.^[^ ^64^, ^65^ ^]^ DFT‐D3 method was used to correct the van der Waals interactions, respectively.^[^ ^66^ ^]^ One layer of Carbon (001) facet was cleaved with a vacuum layer of 20 Å to build the carbon surface model. One graphite nitrogen, pyrrole nitrogen, pyridine nitrogen and NH_2_ radical was constructed in the carbon surface model to build the C‐N‐1 model, C‐H‐2 model, C‐N‐3 model and C‐NH_2_ model, respectively. All models were fully relaxed with the energy convergence criterion of 10^−5^ eV and the force convergence criterion of 0.02 eV Å^−1^, respectively. The (2 × 2 × 1) K‐points were used in the K‐point mesh. The adsorption energy (E_ads_) was calculated using the formula:

(4) $$E_{\mathrm{ads}}=E_{\mathrm{total}}-E_{\mathrm{substrate}}-E_{\mathrm{adsorbate}}$$

The *E*_total_, *E*_substrate_ and *E*_adsorbate_ represent the energy of adsorption structure, substrate and adsorbate, respectively.

### Fabrication of the Pam‐CDs‐Based Photodetector Array

A 6 × 6 pixels electrode array was prepared through thermal evaporation and photolithography. The concentrated *pam*‐CDs aqueous solution was then dropped on the electrode array and allowed to dry naturally.

### Trajectory Recognition Testing Method

The sensor array was placed on a 3D displacement platform, with the platform height adjusted to enable humidity detection from the finger. After fixing the finger's position, relative movement between the finger and the sensor array was achieved using the displacement platform, enabling trajectory recognition.

## Conflicts of interest

The authors declare no conflict of interest.

## Supporting information



Supporting Information

## Data Availability

The data that support the findings of this study are available from the corresponding author upon reasonable request.
