# Supplementary material for: Surface Engineering Enabled Capacitive Gas‐Phase Water Molecule Sensors in Carbon Nanodots
Source: Adv Sci (Weinh). 2025 Apr 26;12(21):2414611. doi: 10.1002/advs.202414611 (PMC12140313; doi:10.1002/advs.202414611)
Supplement: Supplementary file 1 — Supporting Information [file ADVS-12-2414611-s001.docx]

**Supporting Information**

**Surface Engineering Enabled Capacitive Gas-phase Water Molecule Sensors in Carbon Nanodots**

*Jin-Xu Qin, Cheng-Long Shen*, Wu-You Zhang, Yuan Deng, Shou-Long Lai, Chao-Fan Lv, Hang Liu, Ying-Jie, Zhang, Lan Liu, Lei Li, Xi-Gui Yang*, Chong-Xin Shan**

J. X. Qin, C. L. Shen, W. Y. Zhang, S. L. Lai, C. F. Lv, H. Liu, Y. J. Zhang, L. Liu, L. Li, X. G. Yang, C. X. Shan

Henan Key Laboratory of Diamond Optoelectronic Material and Devices, Key Laboratory of Integrated Circuit, Ministry of Education, School of Physics, Zhengzhou University, Zhengzhou 450052, China

X. G. Yang

Institute of Quantum Materials and Physics, Henan Academy of Sciences, Zhengzhou 450046, China

Y. Deng

School of Computational Science and Electronics, Hunan Institute of Engineering, Xiangtan 411104, China

E-mail: phyclshen@zzu.edu.cn (C. L. Shen), yangxg@zzu.edu.cn (X. G. Yang), cxshan@zzu.edu.cn (C. X. Shan)

**Figures**

**
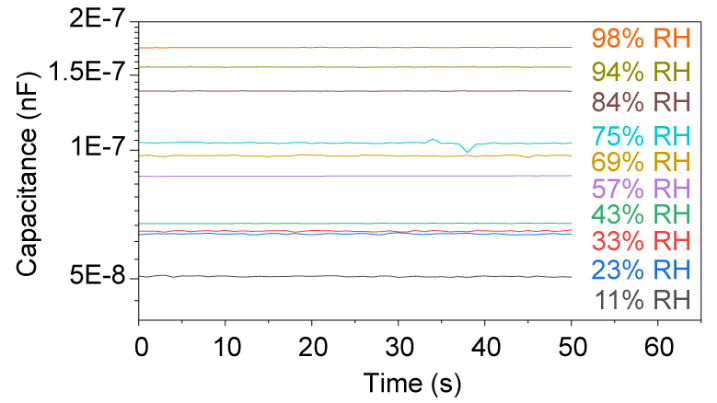
**

**Figure S1.** *C-t* curves of the precursor*s*-based sensor @100 Hz/5V under different RHs.


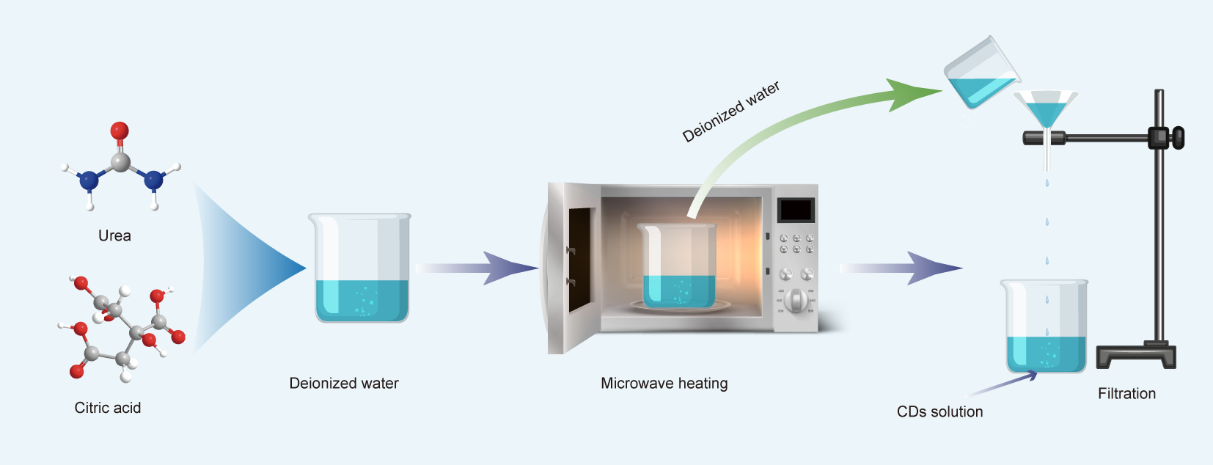


**Figure S2.** Schematic diagram of the synthesis process for CDs.


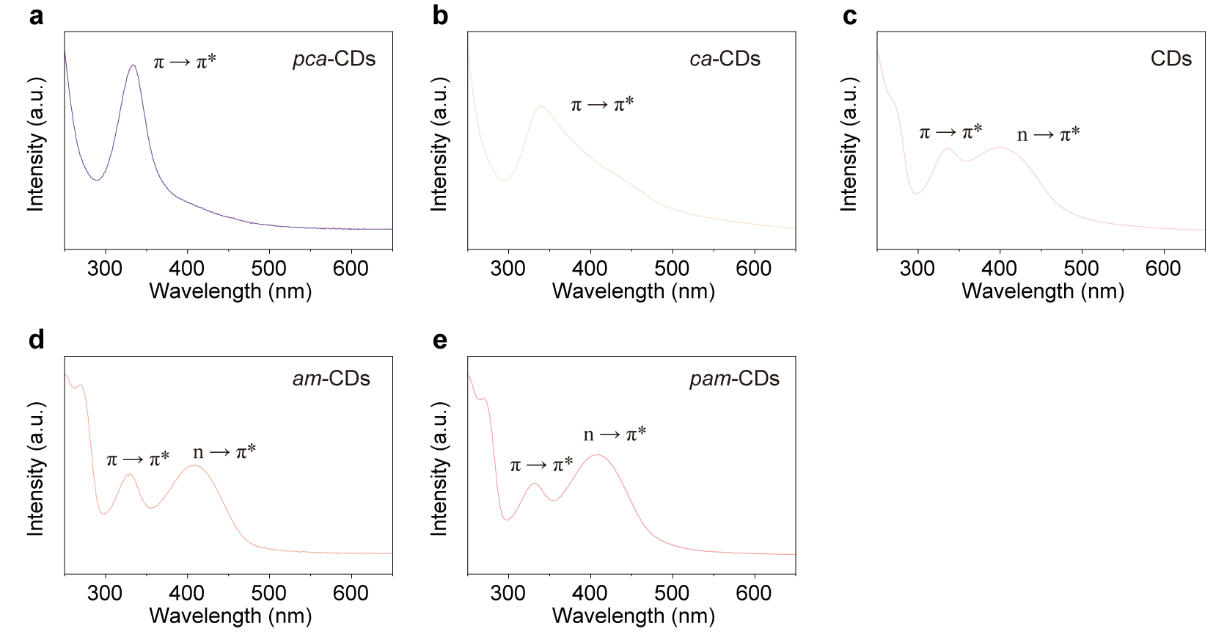


**Figure S3.** (a) UV-vis absorption spectra of the *pca*-CDs solution. (b) UV-vis absorption spectra of the *ca*-CDs solution. (c) UV-vis absorption spectra of the CDs solution. (d) UV-vis absorption spectra of the *am*-CDs solution. (e) UV-vis absorption spectra of the *pam*-CDs solution.


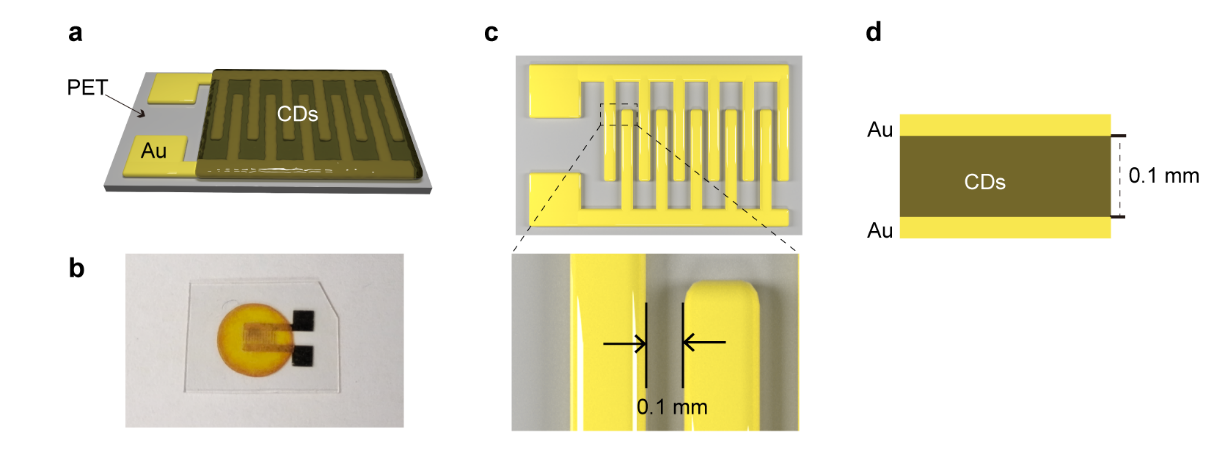


**Figure S4.** (a) Schematic illustration of the structure of the CDs-based capacitive humidity sensor. (b) The photograph of the CDs-based capacitive humidity sensor. (c-d) Electrode and corresponding capacitor structure of the CDs-based humidity sensor.


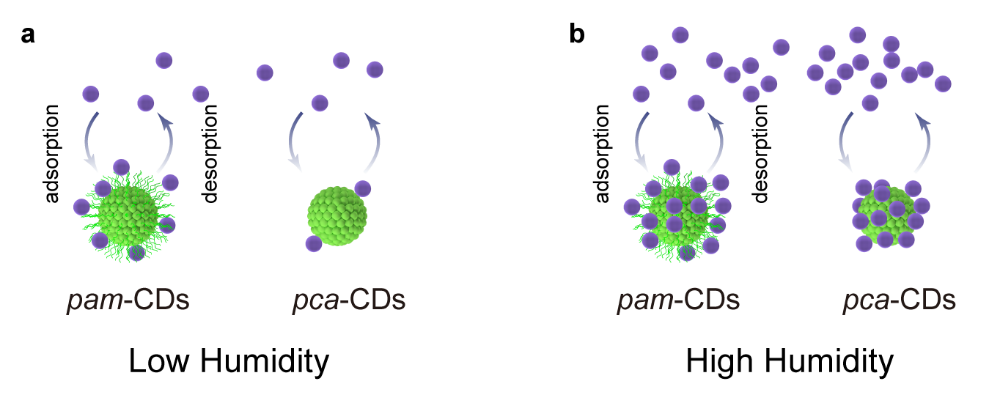


**Figure S5.** (a) Schematic illustration of the adsorption equilibrium of *pam*-CDs and *pca*-CDs at low humidity. (b) Schematic illustration of the adsorption equilibrium of *pam*-CDs and *pca*-CDs at high humidity.


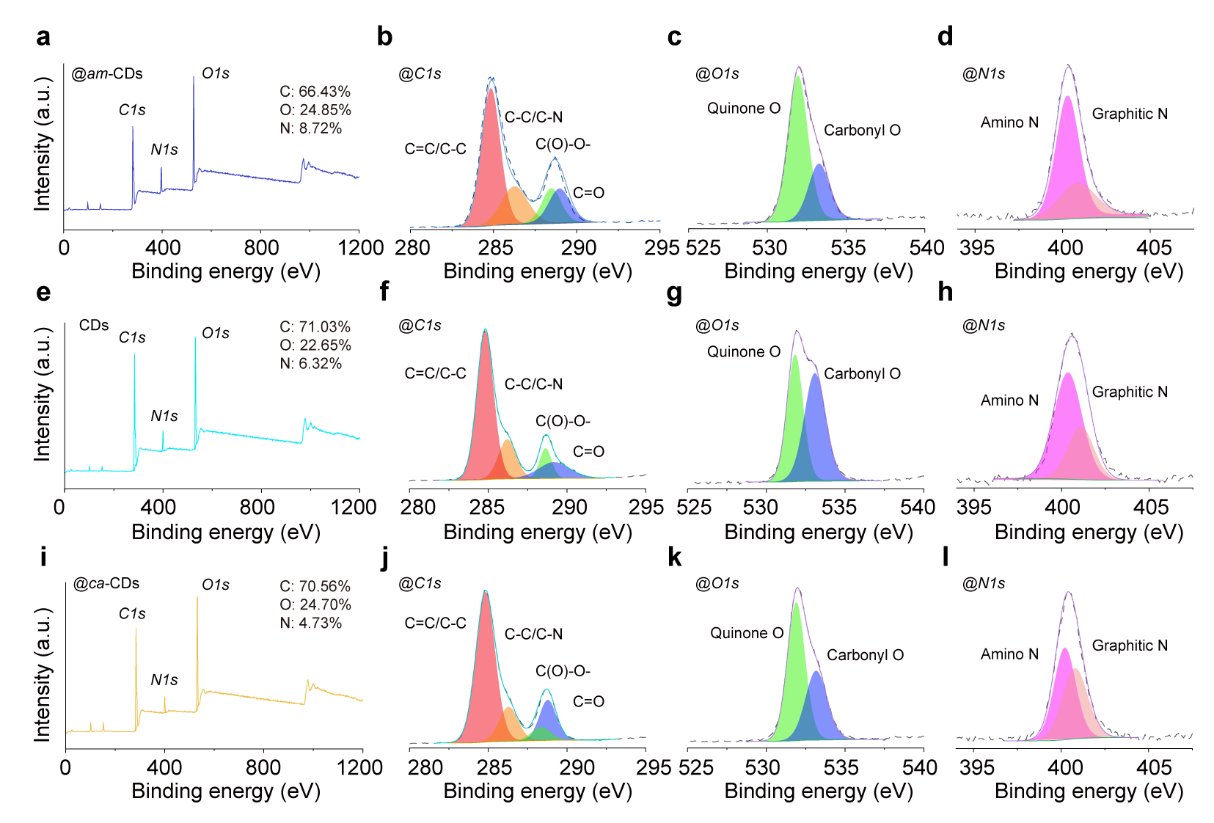


**Figure S6.** a)-d) Full XPS survey (a), high-resolution C1s spectra (b), high-resolution O1s spectra (c) and high-resolution N1s spectra (d) for am-CDs. e)-h) Full XPS survey (e), high-resolution C1s spectra (f), high-resolution O1s spectra (g) and high-resolution N1s spectra (h) for CDs. i)-l) Full XPS survey (i), high-resolution C1s spectra (j), high-resolution O1s spectra (k) and high-resolution N1s spectra (l) for ca-CDs.


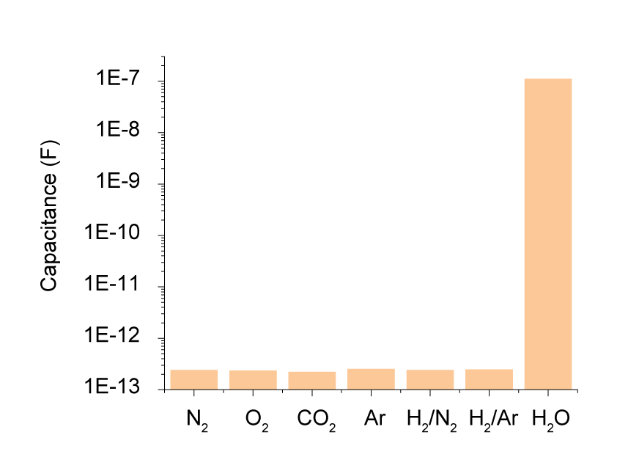


**Figure S7.** Capacitive response of the *pam*-CDs-based sensor in N_2_, O_2_, CO_2_, Ar, H_2_- N_2_ mixture, H_2_-Ar mixture, and H_2_O environment.


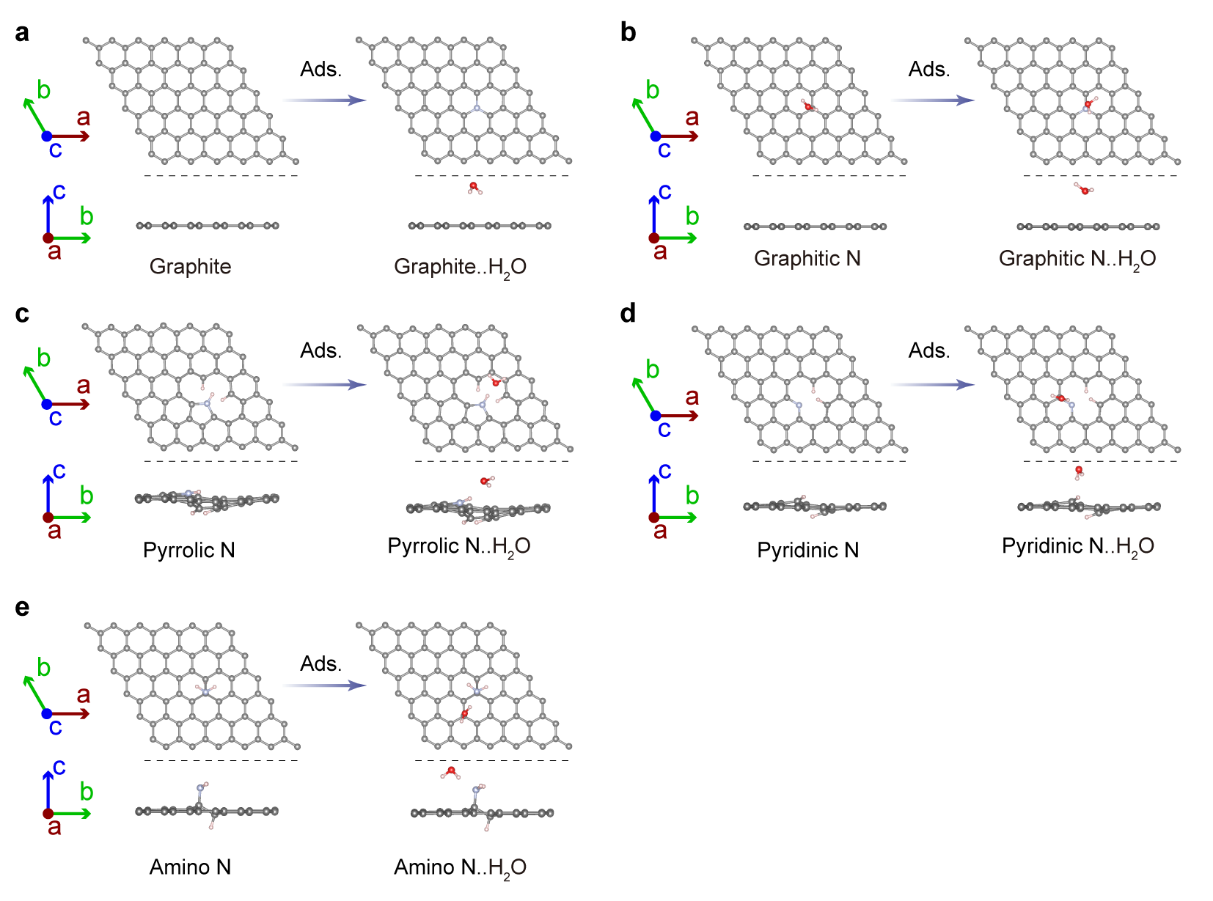


**Figure S8.** a)-e) Schematic illustration of H_2_O molecule adsorption on the surface of graphite (a), graphitic N (b), pyrrolic N (c), pyridinic N (d) and amino N (e).


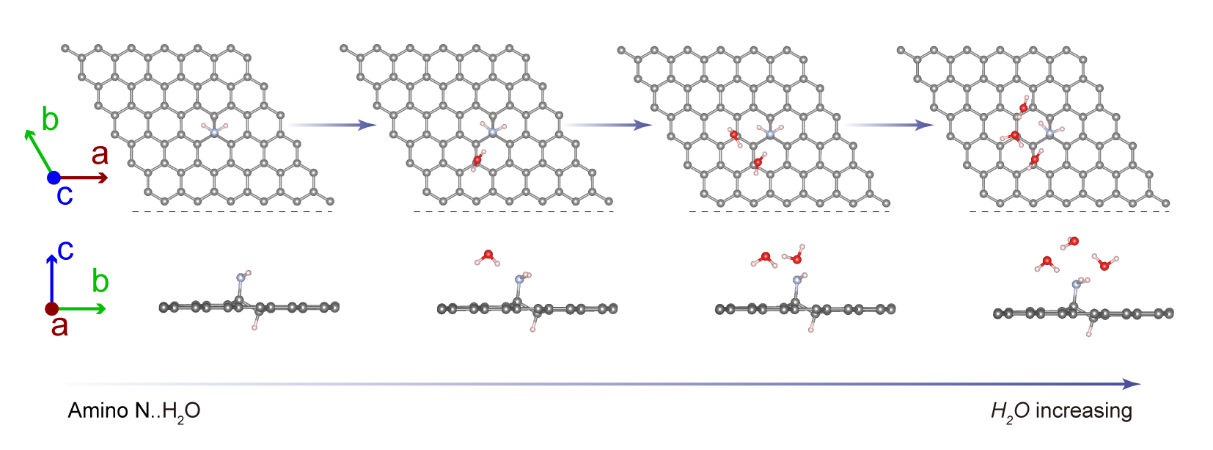


**Figure S9.** Schematic illustration of amino N-related graphite surfaces with 0, 1, 2, and 3 H_2_O molecules.


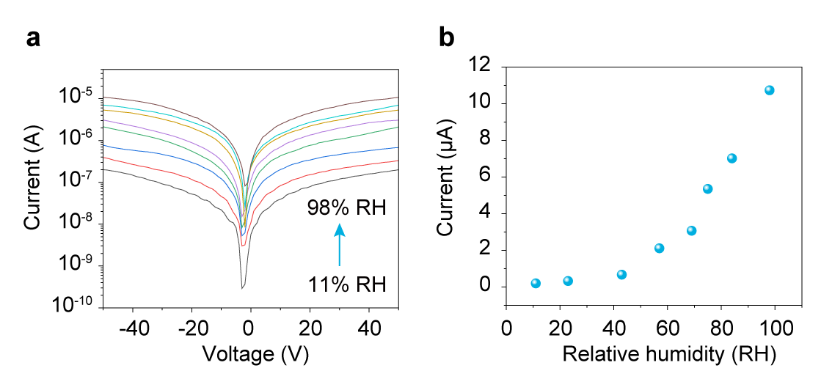


**Figure S10.** (a) Current-Voltage curves of the *pam*-CDs. (b) Current of the *pam*-CDs under different RHs.


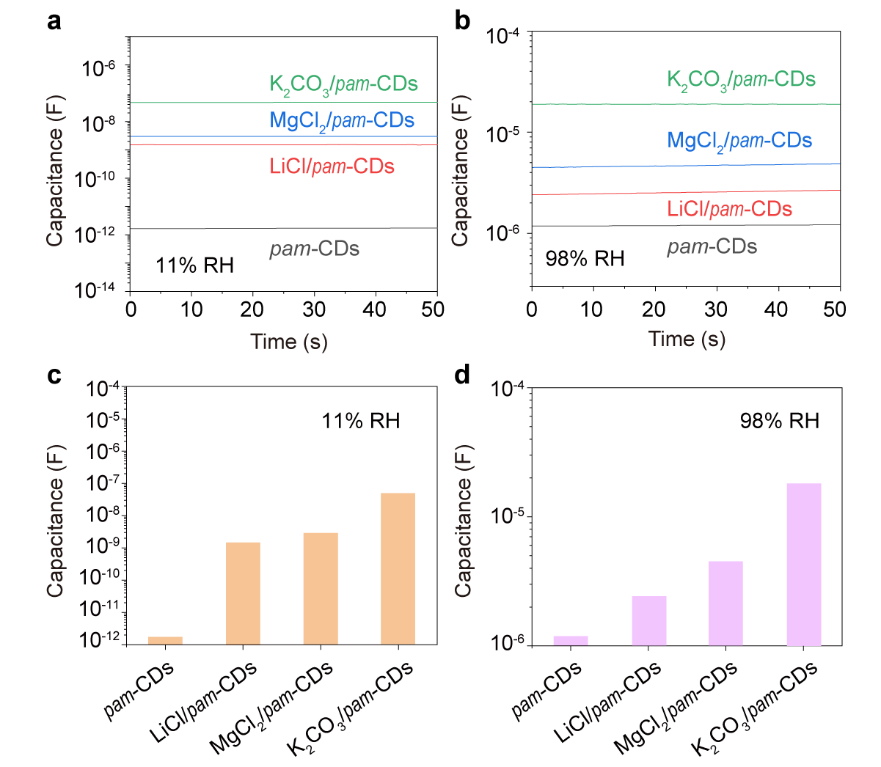


**Figure S11.** (a) *C-t* curves of the *pam*-CDs-based sensor, LiCl/*pam*-CDs-based sensor, MgCl_2_/*pam*-CDs-based sensor and K_2_CO_3_/*pam*-CDs-based sensor under 11% RH. (b) *C-t* curves of the corresponding sensor under 98% RH. (c) Capacitive of the corresponding sensor under 11% RH. (c) Capacitive of the corresponding sensor under 98% RH.


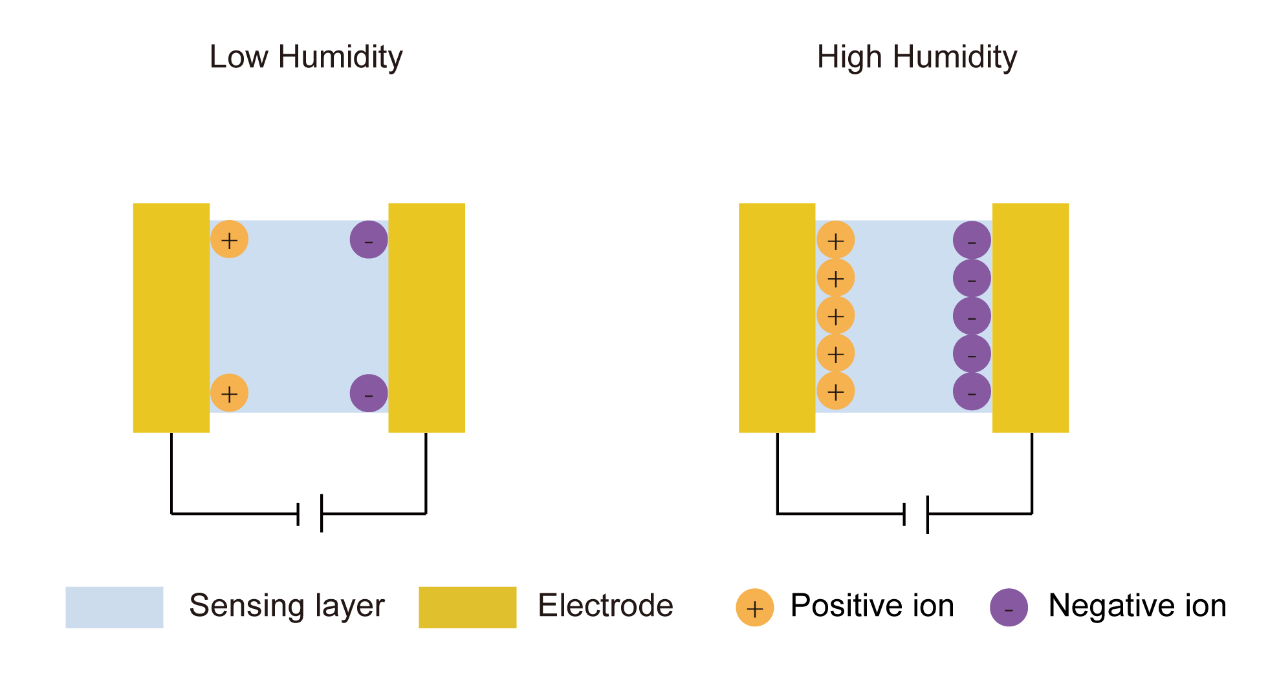


**Figure S12.** The possible EDL effect in the *pam*-CDs-based sensor.


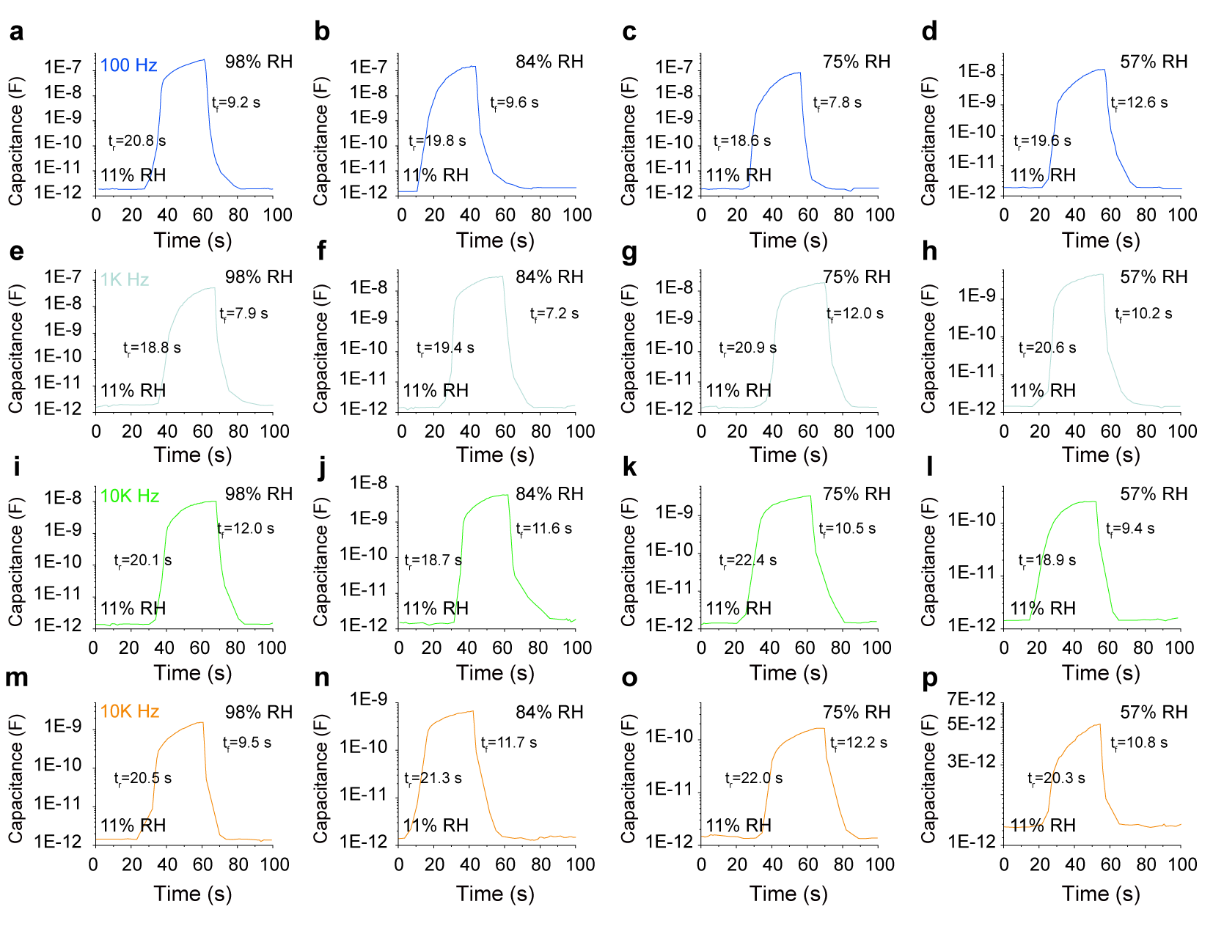


**Figure S13.** (a-d) Response and recovery curves of the CDs-based sensor under different RHs @100 Hz. (e-h) Response and recovery curves of the CDs-based sensor under different RHs @1K Hz. (i-l) Response and recovery curves of the CDs-based sensor under different RHs @10K Hz. (m-p) Response and recovery curves of the CDs-based sensor under different RHs @100K H


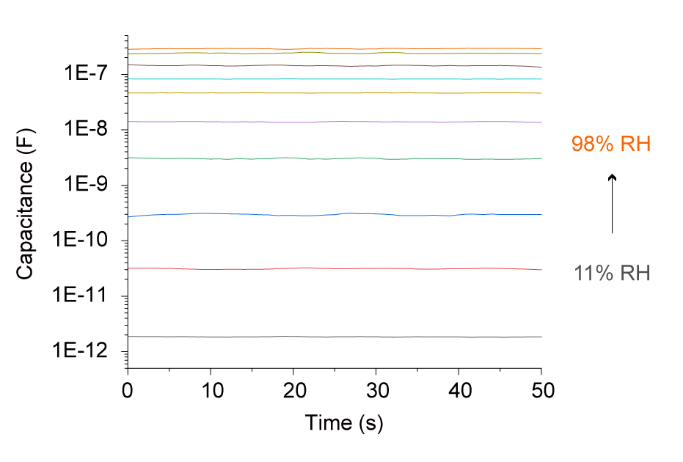


**Figure S14.** *C-t* curves of the pam-CD-based sensor after being placed for 121 days @100 Hz/5V under different RHs.


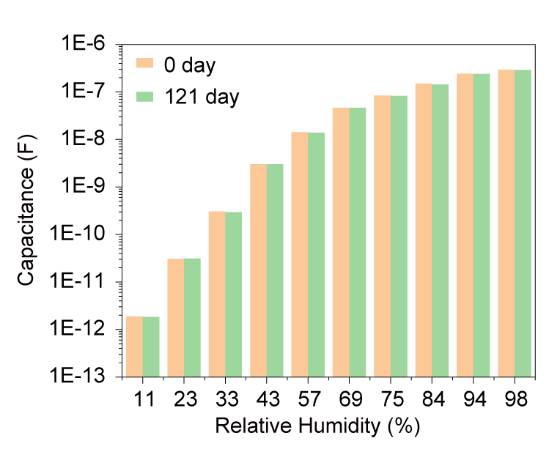


**Figure S15.** Stability of the *pam*-CD-based sensors under different RHs for 121 days.


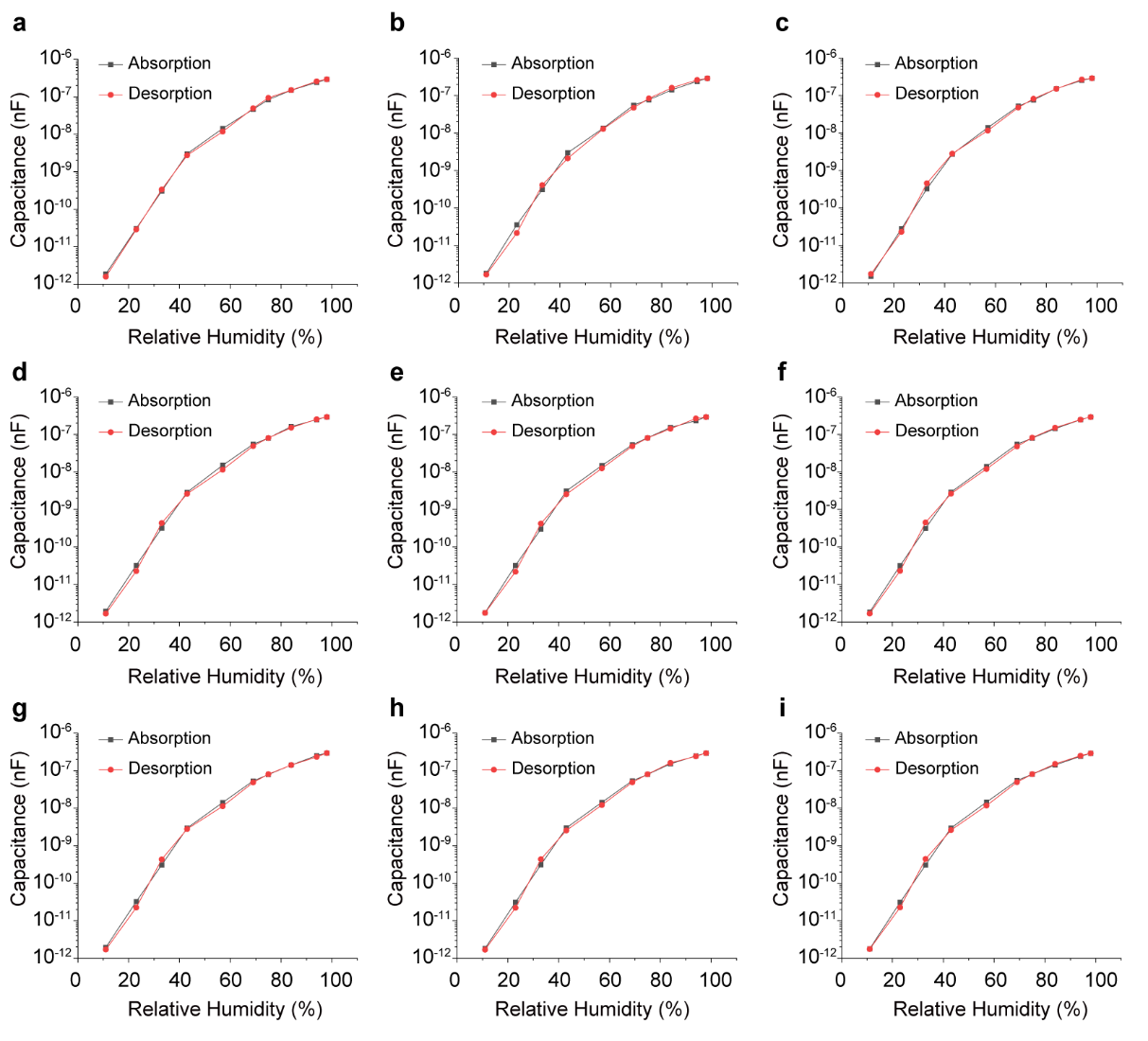


**Figure S16.** (a-i) Humidity hysteresis curves of the sensor in nine tests.


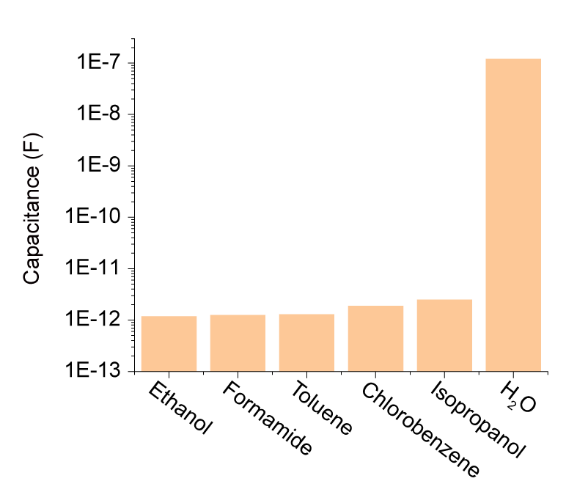


**Figure S17.** Capacitive response of the *pam*-CDs-based sensor in ethanol, formamide, toluene, chlorobenzene, isopropanol, and H_2_O environment.


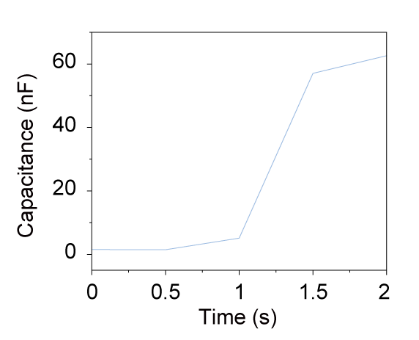


**Figure S18.** Capacitance changes of the sensor during exhalation.


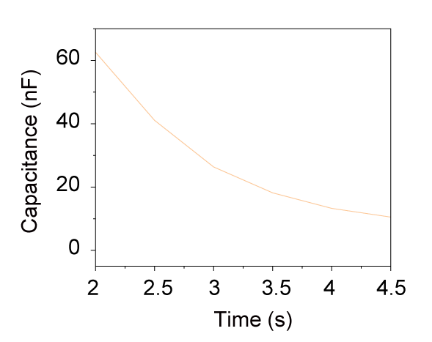


**Figure S19.** Capacitance changes of the sensor during inhalation.


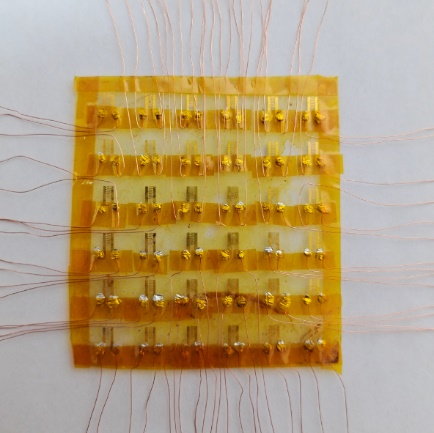


**Figure S20.** Photograph of the *pam*-CD-based sensor array.


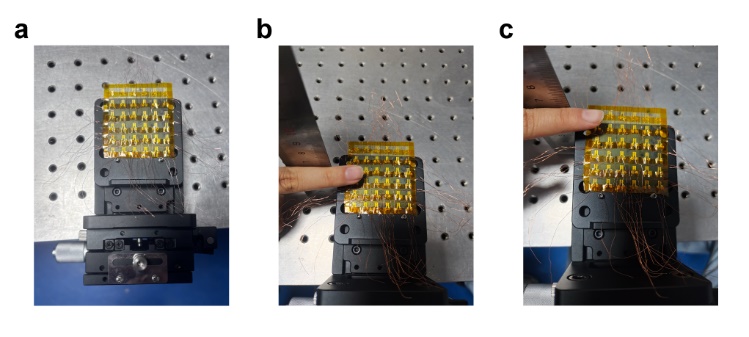


**Figure S21.** (a) Photograph of the *pam*-CD-based sensor array on a displacement platform. (b,c) Finger at different positions on the array.


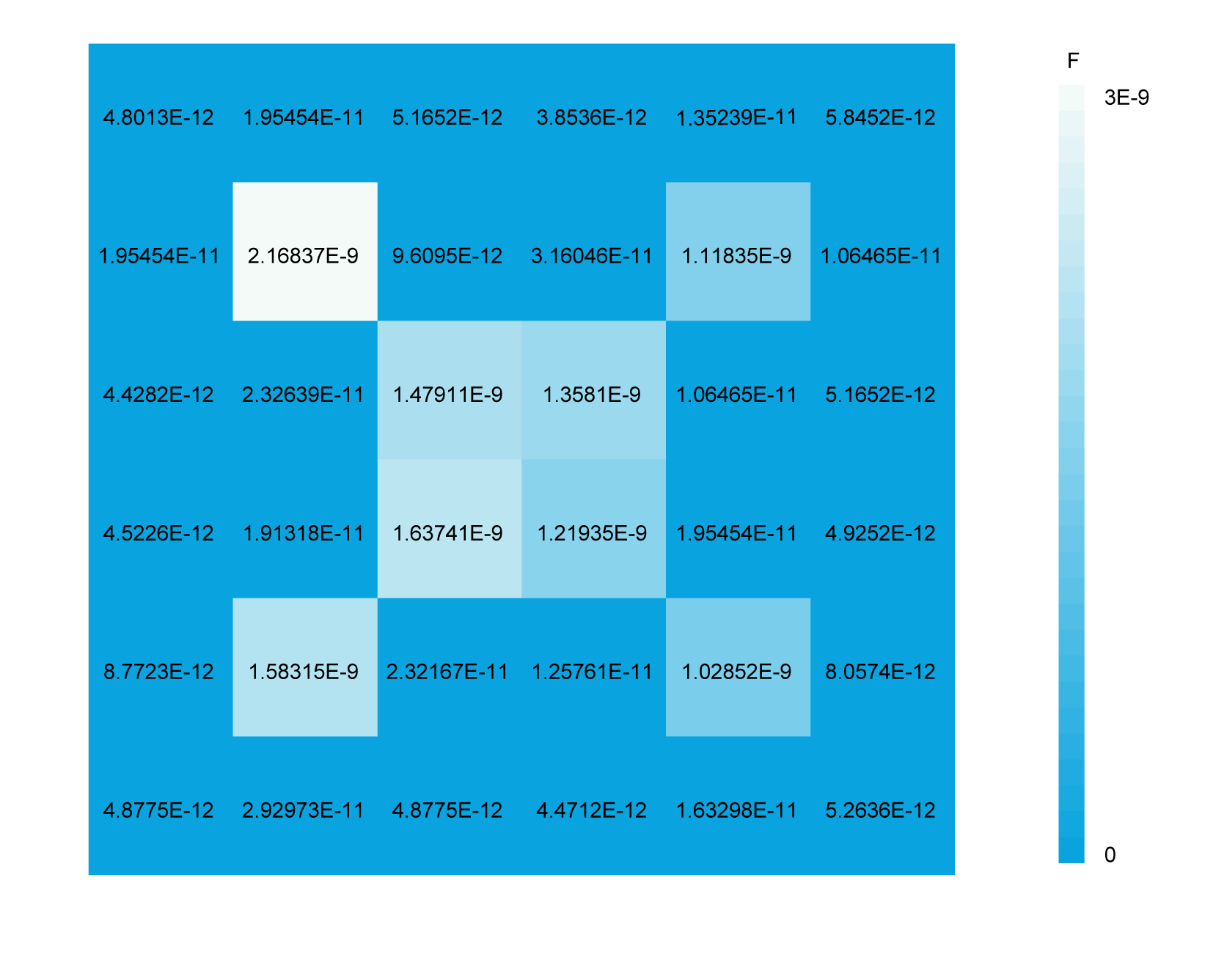


**Figure S22.** Imaging maps and capacitive response of shape “X” by a finger above the array.


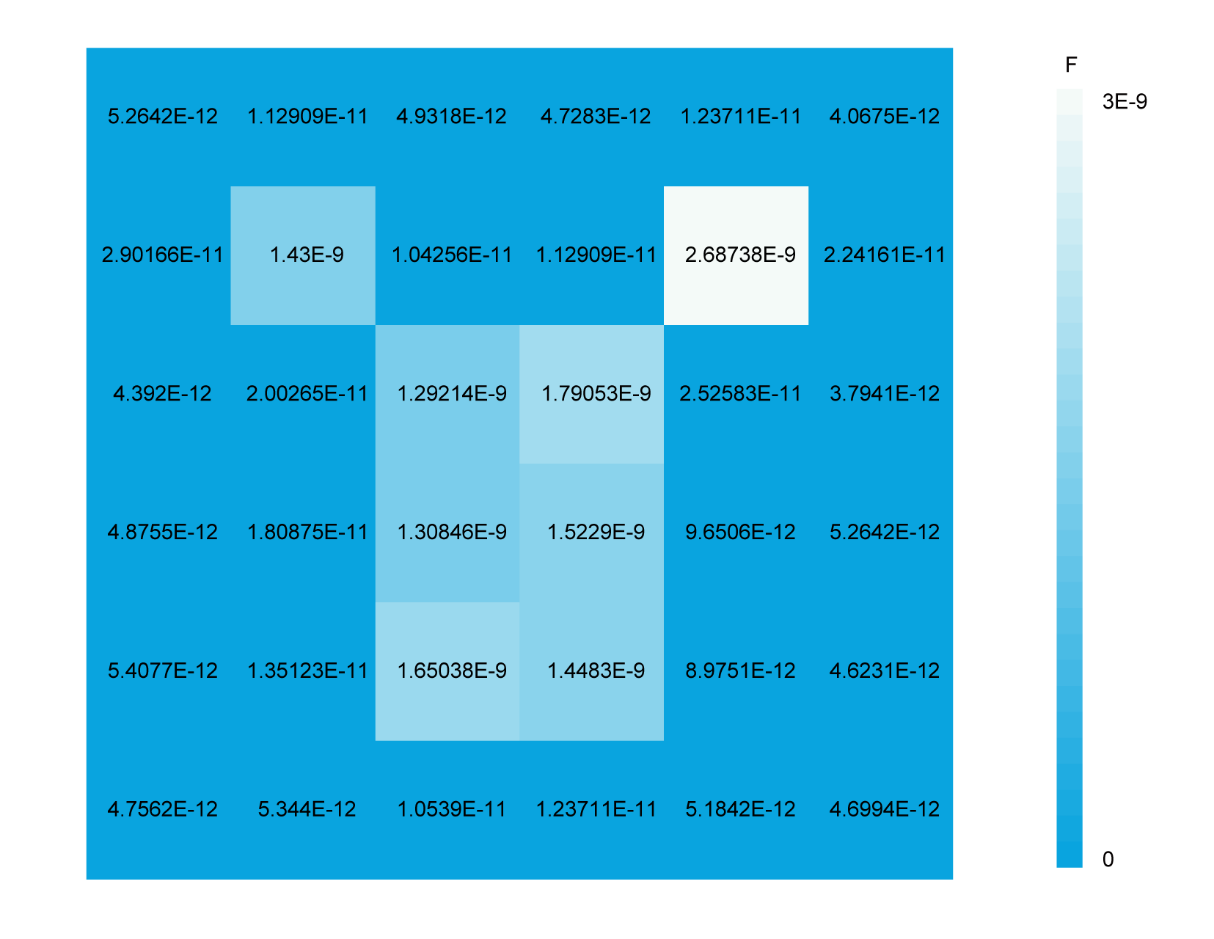


**Figure S23.** Imaging maps and capacitive response of shape “Y” by a finger above the array.


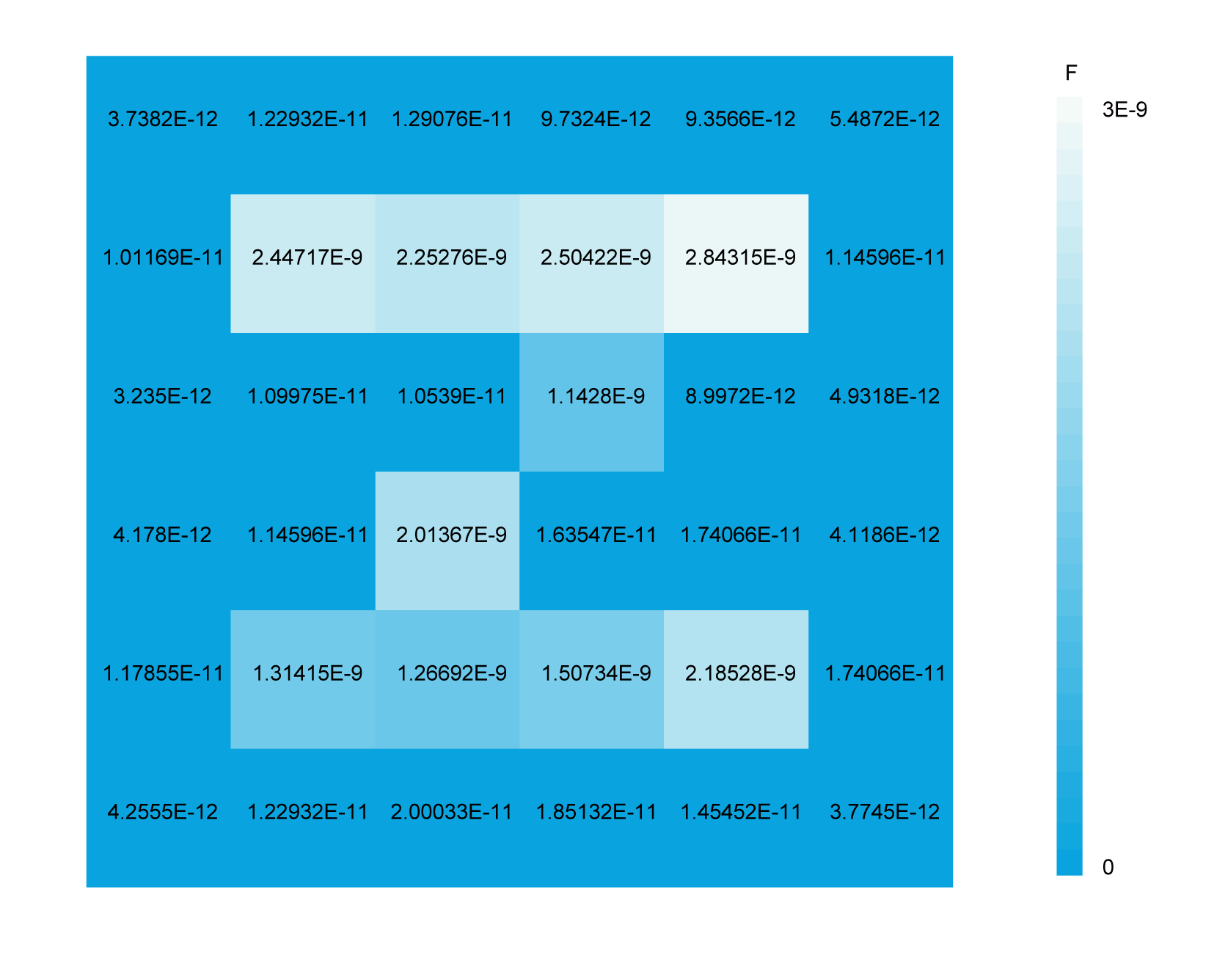


**Figure S24.** Imaging maps and capacitive response of shape “Z” by a finger above the array.


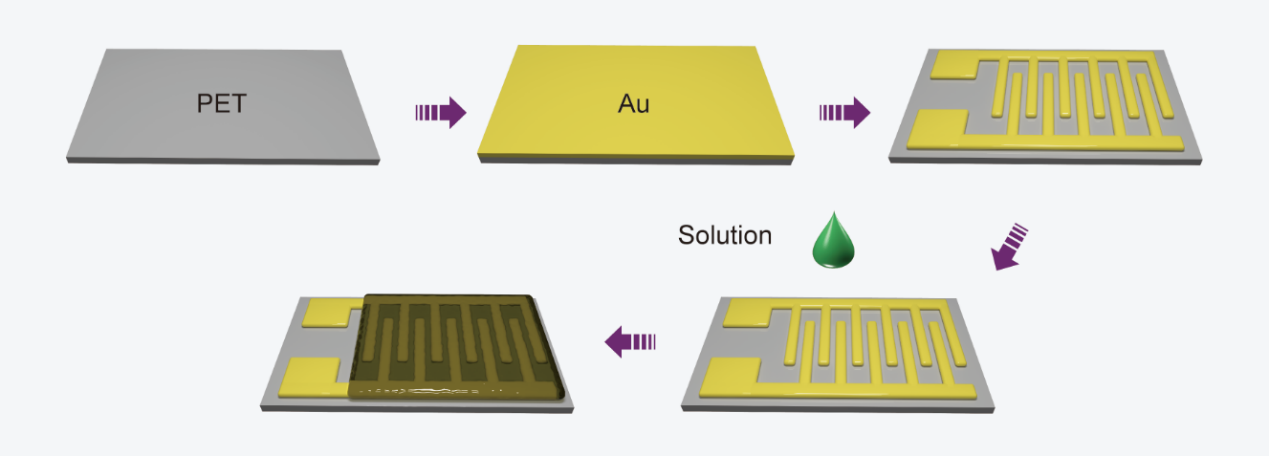


**Figure S25.** Schematic diagram of the sensor fabrication process.
